# Length-Dependent Behavior of Linear dsDNA in IP-RP-HPLC on Supermacroporous Polymer Resin

**DOI:** 10.1021/acs.analchem.6c02096

**Published:** 2026-09-08

**Authors:** Simonas Balčiu̅nas, Audrius Žvirblis, Matas Damonskis, Frank Steiner, Evaldas Naujalis, Lukas Taujenis

**Affiliations:** † Department of Analytical and Environmental Chemistry, Vilnius University, Naugarduko 24, LT-03225 Vilnius, Lithuania; ‡ Thermo Fisher Scientific Baltics, V. A. Graičiu̅no 8, LT-02241 Vilnius, Lithuania; § 125503 Thermo Fisher Scientific, Dornierstraße 4, 82110, Germering, Germany; ∥ State Research Institute Center for Physical Sciences and Technology, Saulėtekio av. 3, LT-10257 Vilnius, Lithuania

## Abstract

Ion-pair reversed-phase high performance liquid chromatography (IP-RP-HPLC) is widely employed for the characterization of therapeutic oligonucleotides and mRNA vaccines. However, the retention behavior of linear double-stranded DNA (dsDNA), especially very large fragments, is not well-defined under IP-RP-HPLC conditions. This work systematically evaluates separations of dsDNA from 35 bp to 20 kbp using a monodisperse supermacroporous polystyrene-divinylbenzene polymer resin. Various parameters, including column length, flow rate, temperature, and ion-pairing reagent were investigated under fixed and varied gradient profiles, to determine their impact on dsDNA retention. A size-dependent behavior emerged, with a practical threshold near 1.5 kbp. Smaller fragments (≤1.5 kbp) exhibited traditional on–off IP-RP-HPLC behavior. Under fixed gradients, higher flow rates together with shorter columns and shallow gradients yielded the highest resolution and peak capacity values. Stronger ion-pairing reagents and elevated temperatures further increased retention and improved resolution in this range. Beyond this length threshold, behavior became less uniform, and the largest fragments (≥5.0 kbp) showed the opposite trend: longer columns, steep gradients, and lower flow rates improved resolution. In contrast to smaller fragments, weaker ion-pairing reagents provided better separations for large dsDNA, with pair-specific optima that depended on temperature and flow. Additionally, very large fragments exhibited measurable retention and resolution even in the absence of IP-RP-driven retention. This supports the presence of a secondary retention mechanism, which is concurrently present during IP-RP-HPLC separation of large dsDNA. These results demonstrate size-dependent duality of dsDNA and provide practical guidelines for IP-RP-HPLC method optimization for both small and large dsDNA molecules.

## Graphical abstract

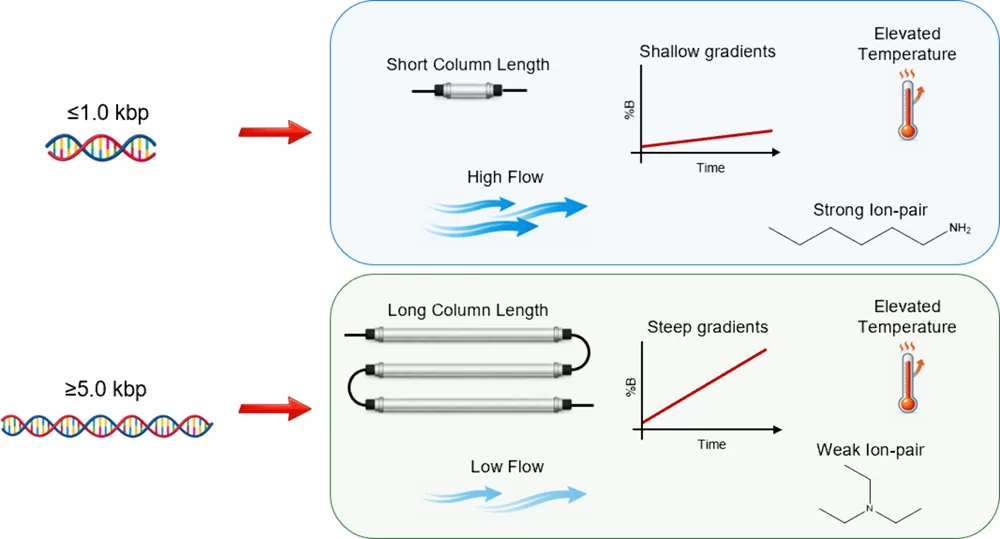

## Introduction

Separation and isolation of small and large double-stranded DNA (dsDNA) is relevant for applications ranging from polymerase chain reaction (PCR) product analysis to DNA restriction mapping, gene therapy and plasmid purification.^1–6^ A variety of analytical techniques can be used to characterize dsDNA fragments, including polyacrylamide and agarose gel electrophoresis (PAGE and AGE),^7,8^ capillary gel electrophoresis (CGE),^9,10^ and various chromatographic approaches.^11^ Among chromatography modes, size-exclusion chromatography (SEC), anion-exchange chromatography (AEX), hydrophilic interaction chromatography (HILIC), and ion-pair reversed-phase high performance liquid chromatography (IP-RP-HPLC) are widely employed for high-resolution separation and purification of nucleic acids (NAs).^12–14^ In addition, slalom chromatography (SC) has recently started regaining interest as a potential approach for resolving large (≥2.0 kbp) dsDNA fragments.^15^

IP-RP-HPLC is commonly used to separate and characterize small oligonucleotide therapeutics (e.g., ASOs, siRNA, sgRNA)^16–18^ and mRNA vaccines.^19,20^ It has also been applied for separation of plasmids and DNA restriction fragment analysis.^1,21^ In IP-RP-HPLC, retention of NAs on hydrophobic stationary phases is enabled by employing positively charged alkylamines that function as ion-pairing reagents (IPRs). Two mechanisms contribute to the retention: formation of a neutral ion-pair complex with NA, and adsorption of the IPR onto stationary phase surface.^22,23^ The relative contribution of each mechanism depends largely on the nature of alkylamine. Weaker IPRs, such as diethylamine and triethylamine, favor ion-pair formation, which increases the hydrophobicity of NAs and facilitates their retention via hydrophobic interactions. Under these conditions, differences in nucleobase composition can contribute more strongly to retention, resulting in greater sequence selectivity. In contrast, stronger, more hydrophobic IPRs (e.g., hexylamine and octylamine) more readily adsorb onto the stationary phase surface, creating a positively charged layer that shifts the retention to be governed primarily by anion-exchange interactions. As the contribution of the ionic retention mechanism increases with increasing IPR hydrophobicity, separations become increasingly length-dependent and less influenced by NA sequence composition.^24–27^ The relationship between sequence- and length-dependent retention for dsDNA may differ from that of single-stranded NAs due to differences in their molecular structure. For single stranded NAs, both the nucleobases and phosphate backbone are readily accessible and can therefore contribute substantially to chromatographic selectivity. In contrast, the double-helical structure of dsDNA reduces accessibility of the nucleobases while the negatively charged phosphate backbone remains exposed to the surrounding solution.^28^ Consequently, interactions involving the phosphate backbone are expected to play a greater role in dsDNA retention, whereas the influence of nucleobase composition on chromatographic selectivity is reduced relative to single-stranded NAs. Additionally, IP-RP workflows for nucleic acid analysis include acidic counterions in mobile phase to control pH and protonate alkylamines. Acetic acid is typically used for ultraviolet (UV) detection, while hexafluoroisopropanol (HFIP) is preferred for mass spectrometric detection because it enhances ionization efficiency and improves electrospray response.^29–31^ Furthermore, HFIP reduces the solubility of alkylamines, promoting greater IPR adsorption on stationary phase surface.^32^ This enhances contribution from ionic interactions and improves length-based separation of NAs. However, HFIP is less commonly chosen for UV-only workflows due to its high cost and reduced mobile phase stability.

IP-RP-HPLC separations employ hydrophobic stationary phases, including silica-based octadecyl (C18) and phenyl,^33^ as well as polymeric polystyrene-divinylbenzene (PS-DVB).^34^ Silica columns for oligonucleotides typically feature smaller pore sizes (120–300 Å), whereas PS-DVB media also offers pore sizes suited for intact analysis of large NAs. Additionally, supermacroporous PS-DVB columns with wide pore size distribution, enable analysis of both large and small NA without sacrificing performance. NAs follow an on–off mechanism: they bind strongly near the column inlet and elute rapidly with a modest increase in mobile phase strength.^35^ Under appropriate chromatographic conditions, this behavior makes short columns highly effective and supports high-throughput workflows,^35,36^ which could aid IP-RP analysis of dsDNA fragments. However, despite widespread use for oligonucleotides and mRNA, IP-RP-HPLC separation of linear dsDNA, particularly very large fragments (≥5.0 kbp), remains underexplored, with previous studies primarily focusing on substantially shorter dsDNA fragments.^21,37,38^

The aim of this study was to investigate the length-dependent retention behavior of linear dsDNA under IP-RP-HPLC conditions and determine how chromatographic parameters influence its separation using a 2.5 μm monodisperse supermacroporous PS-DVB polymer resin. The impact of four parameters, including column length, flow rate, temperature and ion-pairing reagent, was investigated across a broad dsDNA range from 35 bp to 20 kbp. To enable size-based comparison, fragments were organized into four distinct ladders, and their retention behavior was studied using both fixed and varied gradient profiles. Resolution (*R*_s_) and peak capacities (*P*) were evaluated for each ladder to compare how separation performance changes with increasing fragment length. Together, this work demonstrates the length-dependent chromatographic trends for linear dsDNA across a broad size range and provides comprehensive practical guidelines for selecting suitable chromatographic conditions for IP-RP-HPLC method development.

## Experimental Section

### Materials and Reagents

2.0 M triethylamine acetate solution (TEAA, Cat. No. 400771), diethylamine (99+% extra pure, Cat. No. 149452500), *n*-butylamine (99+%, Cat. No. 107800010), ammonium acetate (AmAc, Optima LC/MS grade, Cat. No. A114–50), acetic acid (99.8%, Cat. No. 222140010), and acetonitrile (ACN, Optima LC/MS grade, Cat. No. A955–212) were purchased from Fisher Scientific (Loughborough, UK). Deionized water (18.2 MΩ·cm) was produced in-house using a Barnstead Smart2Pure water purification system (Thermo Electron LED, Langenselbold, Germany).

### Samples

Double-stranded DNA fragments spanning 0.035, 0.050, 0.075, 0.10, 0.20, 0.30, 0.50, 0.70, 1.0, 1.5, 2.0, 2.5, 5.0, 10, 15, and 20 kbp were obtained from Thermo Fisher Scientific Baltics. The GC content of each dsDNA fragment is provided in Supporting Information, Table S1. Fragments were grouped into four ladders according to size: 35–100 bp, 200–700 bp, 1.0–2.5 kbp, and 5.0–20 kbp. Each ladder was prepared by adding 20 μL of individual fragments (0.5 μg/μL) and diluting with deionized water to a final volume of 1 mL, yielding a final concentration of 0.01 μg/μL for each fragment.

### Instrumentation

All experiments were carried out on a biocompatible Vanquish Horizon UHPLC system (Thermo Fisher Scientific) equipped with a binary pump with integrated degasser and 35 μL mixer, an autosampler with a 25 μL sample loop, a column compartment with an active preheater, and a variable-wavelength detector fitted with a semimicro bio 2.5 μL flow cell. Capillaries with a 0.1 mm inner diameter were used in this instrument configuration. Pressure-dependence experiments were carried out with a 75 × 550 μm Viper (Thermo Fisher Scientific) capillary installed as a postcolumn restrictor between column and detector, to increase system backpressure. The instrument was controlled using Chromeleon 7.3.1 software (Thermo Fisher Scientific).

### Chromatography and Methodology

Linear dsDNA separations were performed on SurePac Oligo RP MDi analytical columns (Thermo Fisher Scientific) packed with 2.5 μm monodisperse supermacroporous polystyrene-divinylbenzene polymer particles featuring a broad intraparticle pore size distribution. The 2.1 × 20 mm, 2.1 × 50 mm, and 2.1 × 100 mm columns were used as received, whereas the 2.1 × 200 mm and 2.1 × 300 mm formats were assembled by combining two and three 2.1 × 100 mm columns in series using low-dead volume Viper capillaries (1 μL added volume per single fitting). Unless stated otherwise, all experiments were conducted under identical chromatographic conditions. Mobile phase A was 100 mM TEAA (pH 7.0) and mobile phase B was ACN. A flow rate of 0.2 mL/min was used, with columns maintained at 30 °C and UV detection at 260 nm. The injection volume was 1 μL for all dsDNA ladders. Separations with fixed gradient time (*t*_g_) were performed under linear gradient program from 5%B to 25%B at 2%B per minute, followed by a 2 min wash at 100%B and an 8 min re-equilibration at 5%B. When changes in column length or flow rate required proportional adjustment of gradient profile to keep the gradient retention factor constant (k*), the *t*_g_ was scaled according to eq 1.^39^

1 $$k^{*}=\frac{0.87t_{g}F}{V_{c}\Delta \phi S}$$

where k* is the gradient retention factor, *t*_g_ is the gradient time (min), *F* is the flow rate (mL/min), *V*_c_ is the column volume (mL), Δϕ is change in volume fraction of the mobile phase (%), and *S* is a constant for a given compound under the specified experimental conditions.

Resolution between adjacent dsDNA fragment pairs was calculated according to the USP definition. Peak capacity between the first and last ladder fragments was calculated using the average peak width at half height with eq 2.^24,40^

2 $$P=1+\frac{t_{R\left(n\right)}-t_{R\left(1\right)}}{1.7\times w_{50\%}}$$

where *P* is peak capacity, *t*_*R*(*n*)_ and *t*_*R*(1)_ are retention times for last and first fragments within the ladder (min), and *w*_50%_ is the average peak width at half height (min).

### Stationary Phase Characterization

The morphology and porosity of SurePac Oligo RP MDi PS-DVB particles were characterized using scanning electron microscopy (SEM), mercury intrusion porosimetry (MIP), and nitrogen adsorption porosimetry. SEM imaging was performed using a Hitachi SU3500 scanning electron microscope (Hitachi High-Tech Corporation), MIP measurements were performed using an AutoPore IV 9500 mercury intrusion porosimeter (Micromeritics Instrument Corporation), and nitrogen adsorption porosimetry was performed using an ASAP 2420 surface area and porosity analyzer (Micromeritics Instrument Corporation).

## Results and Discussion

### dsDNA Pore Accessibility

To estimate the accessible pore size range of the stationary phase and compare it with the dimensions of the dsDNA fragments investigated in this study, additional characterization was performed using SEM, MIP, and nitrogen adsorption porosimetry. SEM imaging revealed that the monodisperse supermacroporous PS-DVB particles possess a rough, hierarchical, popcorn-like morphology composed of fused submicrometer polymer domains (Figure S1). This open and irregular structure suggests the presence of both interparticle voids within the packed bed and an interconnected intraparticle-pore network.

To further characterize the pore structure, MIP and nitrogen adsorption porosimetry were performed (Figure S2). During mercury intrusion, the largest voids are filled first. Therefore, the low-pressure intrusion region may include contributions from both interparticle voids and the largest accessible intraparticle pores. Because these contributions cannot be unambiguously separated for particles with such an open morphology, the low-pressure intrusion region was excluded from pore size analysis. This approach minimizes contributions from interparticle voids but may also remove part of the largest accessible intraparticle pore volume. Therefore, nitrogen adsorption porosimetry was used primarily to confirm the presence of smaller pores, whereas MIP was used as the principal indicator of the accessible macropore range. The combined porosimetry data indicate an apparent accessible pore size distribution extending from approximately 20 to 4000 Å. However, the main pore volume contribution is associated with pores larger than approximately 1000 Å. The reported distribution should therefore be regarded as an operationally accessible pore size range rather than an exact geometric pore size distribution.

To relate the measured pore-size distribution to dsDNA accessibility, the characteristic dimensions of the investigated dsDNA fragments were estimated using gyration radius (*R*_g_), which is linked to linear dsDNA size.^41^ *R*_g_ can be derived from dsDNA contour length (*L*_c_), using eq 3.^42^

3 $$R_{g}=0.116L_{c}^{0.57}$$

where *R*_g_ is gyration radius (μm) and *L*_c_ is contour length (μm).

Estimated *R*_g_ values ranged from approximately 93 Å for the 35 bp fragment to 3459 Å for the 20 kbp fragment (Table S2). Compared with the measured pore size distribution of the stationary phase, these estimates suggest that accessibility to the pores should decrease progressively with increasing dsDNA length. Smaller dsDNA fragments are expected to access a larger fraction of the available pore volume, whereas larger fragments should become increasingly excluded from smaller pores and therefore be restricted primarily to the largest pores and interparticle void volume.

Under chromatographic flow conditions, dsDNA molecules experience shear-induced stretching and adopt partially extended conformations.^43,44^ Although their contour length increases, the effective diameter of dsDNA molecules is expected to decrease relative to that of equilibrium coil structures, thereby reducing steric exclusion. Consequently, shear-induced stretching should partially compensate for the size-based exclusion. However, pore accessibility is still expected to decrease with an increasing dsDNA fragment length.

### Column Length

To evaluate the effect of column length on IP-RP-HPLC separation of dsDNA fragments using supermacroporous particles, five column lengths (20, 50, 100, 200, and 300 mm) were tested with four dsDNA ladders.

Two types of experiments were performed with each ladder. In the first, a method developed on the 2.1 × 50 mm column was scaled to the remaining column formats by adjusting *t*_g_ in proportion to column volume. Increasing the column length with proportional *t*_g_ adjustments improved resolution and peak capacity across all dsDNA ladders (Figure 1A). This outcome is expected, because extended gradients generally improve separation performance, regardless of the column length.^39^ The shorter ladders (35–100 and 200–700 bp) showed near-complete baseline resolution for most column formats. Increasing column length from 20 to 300 mm improved *P* values from 10 to 49 for the 35–100 bp ladder and from 6 to 26 for the 200–700 bp ladder, resulting in an over 4-fold gain for both. Major improvement was also observed for the larger ladders (1.0–2.5 and 5.0–20 kbp). The ultrashort column (2.1 × 20 mm) provided little to no separation for either large ladder, whereas extending the column length to 300 mm substantially improved the resolution, with *P* reaching 6 for the 1.0–2.5 kbp ladder. For the 5.0–20 kbp ladder, the 2.1 × 300 mm column format achieved baseline resolution (*R*_s_ = 3.2) for the 5.0 kbp fragment from the rest of the ladder, although remaining large-sized fragments still partially coeluted. Overall, all four ladders followed expected behavior when *t*_g_ was adjusted proportionally to column length.

**Figure 1:**
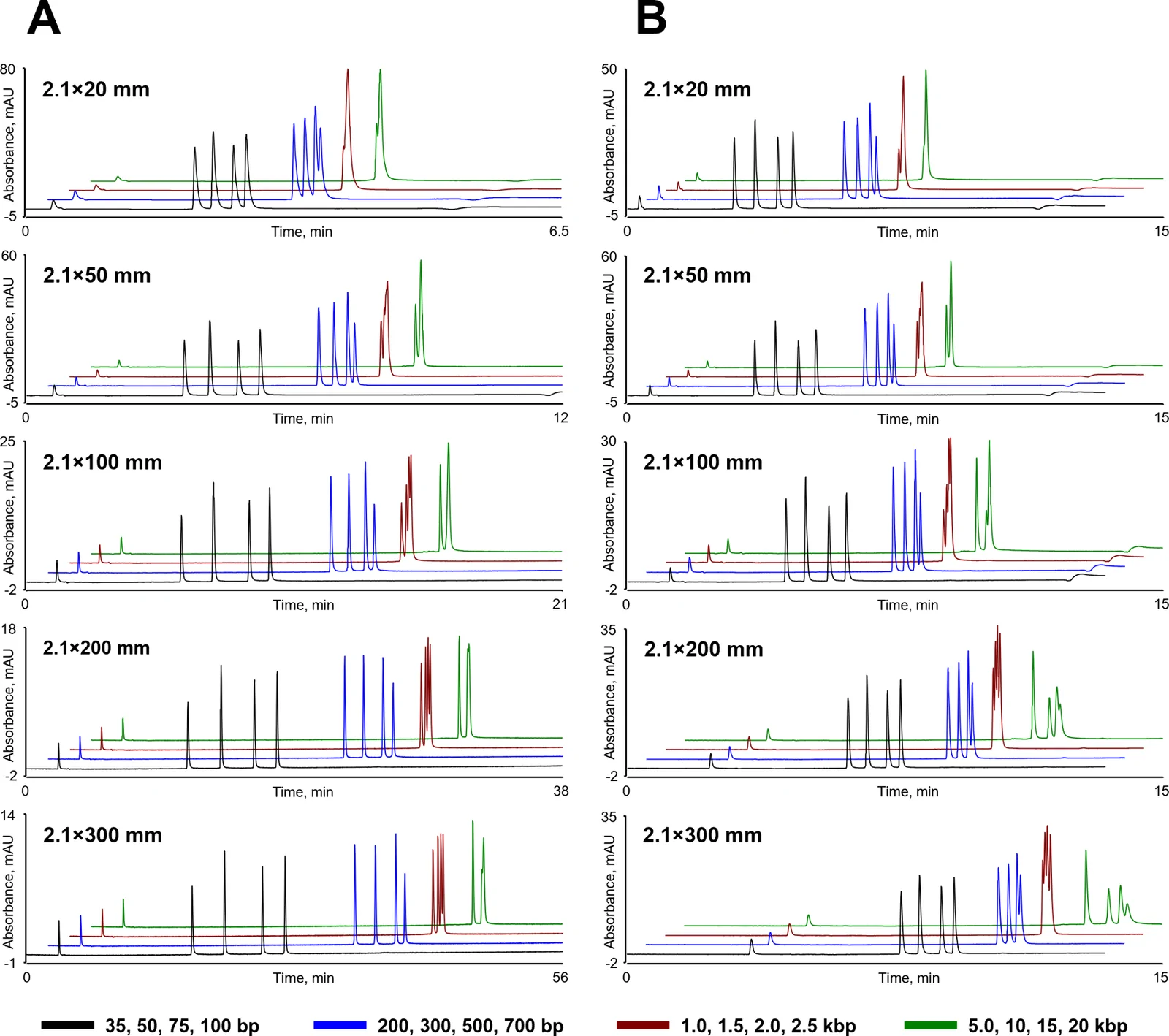
Column length influence on IP-RP-HPLC separation of linear dsDNA fragments ranging from 35 bp to 20 kbp, with gradient times proportionally adjusted to match column length (A), and with fixed gradient profiles (B). Chromatograms were obtained with column lengths of 20, 50, 100, 200, and 300 mm (2.1 mm inner diameter). Black and blue chromatograms represent 35–100 and 200–700 bp, while brown and green represent 1.0–2.5 and 5.0–20 kbp dsDNA ladders. Mobile phase A was 100 mM TEAA (pH 7.0), and mobile phase B was ACN. The injection volume was 1 μL, flow rate was 0.2 mL/min, column temperature was maintained at 30 °C, and UV detection was performed at 260 nm. A linear gradient from 5%B to 25%B was used for both experiments. Gradient times for scaled gradient separations were adjusted relative to 2.1 × 50 mm column format, whereas fixed gradient experiments were performed at 2%B per minute. All dsDNA fragments eluted in order of increasing fragment length.

In the second experiment, the same method developed on the 2.1 × 50 mm column was applied to all column lengths without adjusting the *t*_g_. Under a fixed gradient profile, reducing column length is expected to improve *P* and *R*_s_, because shorter columns have a lower column volume, which is inversely related to peak capacity.^36^ Consequently, increasing column length should diminish separation of dsDNA fragments. As shown in Figure 1B, the 35–100 and 200–700 bp ladders followed this expectation: increasing column length from 20 to 300 mm reduced *P* values from 21 to 15 for 35–100 bp, and from 12 to 7 for 200–700 bp ladder. In contrast, the larger ladders deviated from the expected behavior. Reducing the column length down to 20 mm led to a complete coelution for the 5.0–20 kbp ladder, whereas increasing the length to 300 mm with constant *t*_g_ yielded substantial gains in resolution: all four fragments became clearly distinguishable (*R*_s_ of 5.3, 2.3 and 1.0), with peak capacity of 8. The 1.0–2.5 kbp ladder, however, exhibited a mixed response to increased column length. With the 2.1 × 20 mm column, 1.5, 2.0, and 2.5 kbp fragments coeluted, while the 1.0 kbp fragment was still clearly identifiable (*R*_s_ = 1.1). Increasing column length improved resolution among 1.5–2.5 kbp fragments, but 1.0 and 1.5 kbp fragments separation was reduced. *R*_s_ and *P* values for all ladders and column formats are presented in Supporting Information, Tables S3 and S4.

Prior studies have shown that pressure significantly impacts nucleic acid retention.^45^ At 0.2 mL/min flow rate, extending the column length from 100 to 300 mm resulted in a 3-fold increase in system backpressure (from 155 to 460 bar), which could have potentially influenced dsDNA chromatographic behavior. To test this, all ladders were reanalyzed on a 2.1 × 100 mm column with a postcolumn restriction capillary installed to match the backpressure of the 2.1 × 300 mm format (Figure S3). Apart from a uniform increase in retention across all ladders, no additional changes in separation were observed with the restrictor installed, indicating that higher pressure did not alter selectivity.

Furthermore, during the linear gradient employed in this study, the concentration of IPR decreased as the organic modifier fraction increased. Consequently, smaller dsDNA fragments are separated at higher TEAA concentrations than larger fragments, which could potentially influence the retention mechanism governing the separation, with retention becoming increasingly sequence-dependent as the IPR concentration decreases.^46^ To evaluate this, separations were performed using 100 mM TEAA in both mobile phases A and B with 20, 100, and 300 mm columns (Figure S4). Under constant TEAA concentration conditions, a modest increase in retention and resolution was observed for the 35–100 bp and 200–700 bp ladders. For the larger dsDNA ladders, only a modest increase in retention was observed, while resolution remained largely unchanged compared to separations performed with TEAA present only in mobile phase A. Overall, the same retention and resolution trends were observed under both mobile-phase conditions, indicating that the decrease in the TEAA concentration during gradient elution does not alter the conclusions of the study.

Interestingly, the results presented in Figure 1A,B also suggest that steeper gradients improve separation of large dsDNA fragments. To further investigate this observation, additional experiments were performed using the 5.0–20 kbp ladder on the 2.1 × 300 mm column with gradient times of 5, 10, 30, and 60 min (Figure 2). Consistent with the observation, separation performance improved as gradient time decreased, with the 5 min gradient providing the highest resolution. The improved separation observed under steeper gradients could suggest that prolonged interaction of large dsDNA molecules with the stationary phase becomes detrimental to separation. To investigate this possibility, an additional experiment was performed using a 5 min gradient preceded by a 5 min isocratic hold period. Under these conditions, resolution remained unchanged relative to the 5 min gradient alone (Figure S5). This observation indicates that simply increasing the residence time of dsDNA molecules within the column is insufficient to explain the improved performance obtained with steeper gradients. Instead, the results suggest that events occurring during elution, potentially related to the release of large dsDNA molecules from the stationary phase, may play a more important role.

**Figure 2:**
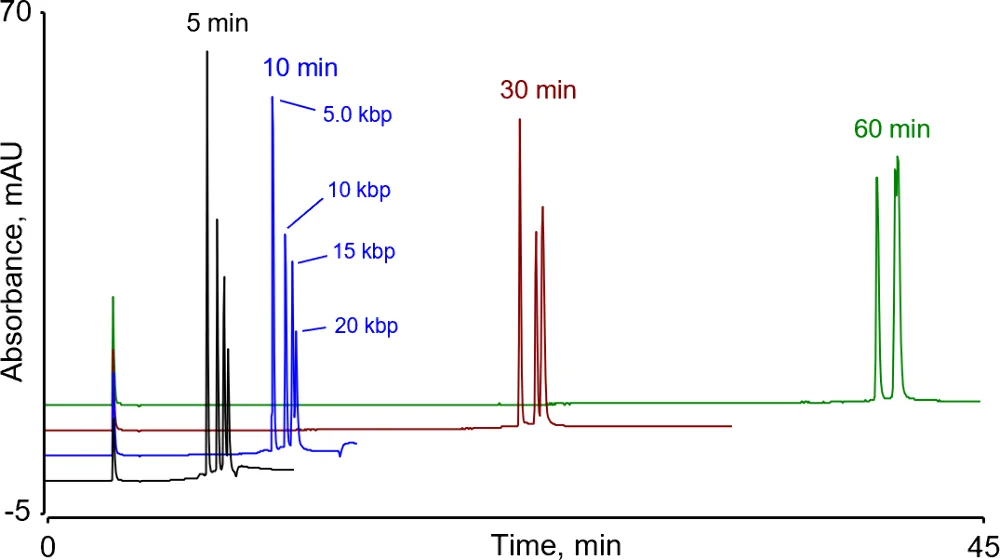
Effect of gradient steepness on IP-RP-HPLC separation of the 5.0–20 kbp dsDNA ladder using a 2.1 × 300 mm column. Chromatograms were acquired using linear gradients from 5%B to 25%B with gradient times of 5, 10, 30, and 60 min. Mobile phase A was 100 mM TEAA (pH 7.0), mobile phase B was ACN, the flow rate was 0.2 mL/min, the column temperature was maintained at 30 °C, the injection volume was 1 μL, and UV detection was performed at 260 nm.

Taken together, the observation that large dsDNA fragments respond differently to changes in column length and gradient steepness indicates a transition in chromatographic behavior occurring between approximately 1.0 and 1.5 kbp. While dsDNA fragments up to approximately 1.0 kbp follow the expected behavior, larger fragments exhibit trends that cannot be readily explained by conventional IP-RP-HPLC on–off retention alone. Instead, the results suggest the presence of an additional size-dependent contribution that becomes increasingly important with increasing dsDNA length and is particularly evident for fragments ≥ 5.0 kbp. This threshold aligns with recent observations of dual retention mechanisms during AEX separations of large linear dsDNA fragments by F. Gritti et al.,^47^ suggesting that secondary retention behavior may also contribute under IP-RP-HPLC conditions.

Given the popcorn-like supermacroporous structure of the particles (Figure S1), one possible explanation for this behavior is the contribution of a secondary obstacle-navigation retention mechanism alongside conventional IP-RP-HPLC retention. In such a case, retention of large dsDNA molecules is governed not only by IP-mediated interactions with the stationary phase but also by a size-dependent steric and topological contribution associated with transport through the supermacroporous stationary phase. Because the effective size of dsDNA increase substantially with fragment length (Table S2), larger molecules are expected to encounter a greater number of pore constrictions and interparticle obstacles along their path. Consequently, longer columns provide a greater distance, potentially enhancing the size-dependent selectivity among large dsDNA fragments.

The benefit of steeper gradients for the 5.0–20 kbp ladder further supports the presence of such a secondary contribution. Under purely IP-RP-HPLC retention, shallower gradients would typically be expected to improve the resolution. However, the opposite trend observed in this study suggests that the separation of large dsDNA is also influenced by processes occurring during elution. One possibility is that large dsDNA fragments undergo a more complex release process as they transition from the retained to mobile state. Shallower gradients prolong the time spent within this transition window, potentially reducing the apparent selectivity generated during transport through the column. In contrast, steeper gradients shorten the duration of this transition and may, therefore, better preserve the size-dependent selectivity arising from the obstacle-navigation retention mechanism.

Moreover, the data demonstrates that ultrashort columns are unsuitable for separations of large dsDNA (≥1.5 kbp), despite substantial differences in fragment length. By contrast, shorter dsDNA fragments follow the traditional on–off IP-RP-HPLC behavior without apparent duality. Therefore, ultrashort columns can be successfully applied to dsDNA fragments up to roughly 1.0 kbp, similarly as other groups have used such columns for separating oligonucleotides of various length.^35^

### Flow Rate

The influence of flow rate on IP-RP-HPLC separation of dsDNA was investigated on 2.1 × 100 mm column at four different flow rates: 0.05, 0.1, 0.2, and 0.4 mL/min. Again, two experimental approaches were used: (i) flow rate was varied with *t*_g_ adjusted proportionally, and (ii) flow was varied with fixed *t*_g_ (no changes in gradient profile).

When *t*_g_ was adjusted to match changes in flow rate (Figure 3A), the 35–100 bp and 200–700 bp ladders showed comparable trends. Higher flow produced sharper peaks, whereas lower flow rates led to broader peaks and slightly increased tailing. Despite that, lowering the flow rate to 0.1 mL/min yielded the highest *P* values (20 for 35–100 bp and 13 for 200–700 bp). However, further reducing the flow rate to 0.05 mL/min led to worse performance due to stronger broadening and pronounced tailing. The 5.0–20 kbp ladder benefited more from decreasing flow when compared to the short ladders. At 0.4 mL/min, baseline separation was achieved only for the 5.0 kbp fragment (*R*_s_ = 2.0), with remaining fragments coeluting. Reducing the flow rate progressively improved separation, and at 0.05 mL/min the overall chromatographic profile was best (*P* = 8), with *R*_s_ values of 4.6, 2.4, and 1.1 among the large-size dsDNA fragments. The 1.0–2.5 kbp ladder showed a mixed pattern: higher flow improved separation among 1.5, 2.0, and 2.5 kbp fragments, whereas lower flow rates worsened it. The 1.0 and 1.5 kbp pairs, by contrast, followed the short ladder trend, with 0.1 mL/min producing the best results.

**Figure 3:**
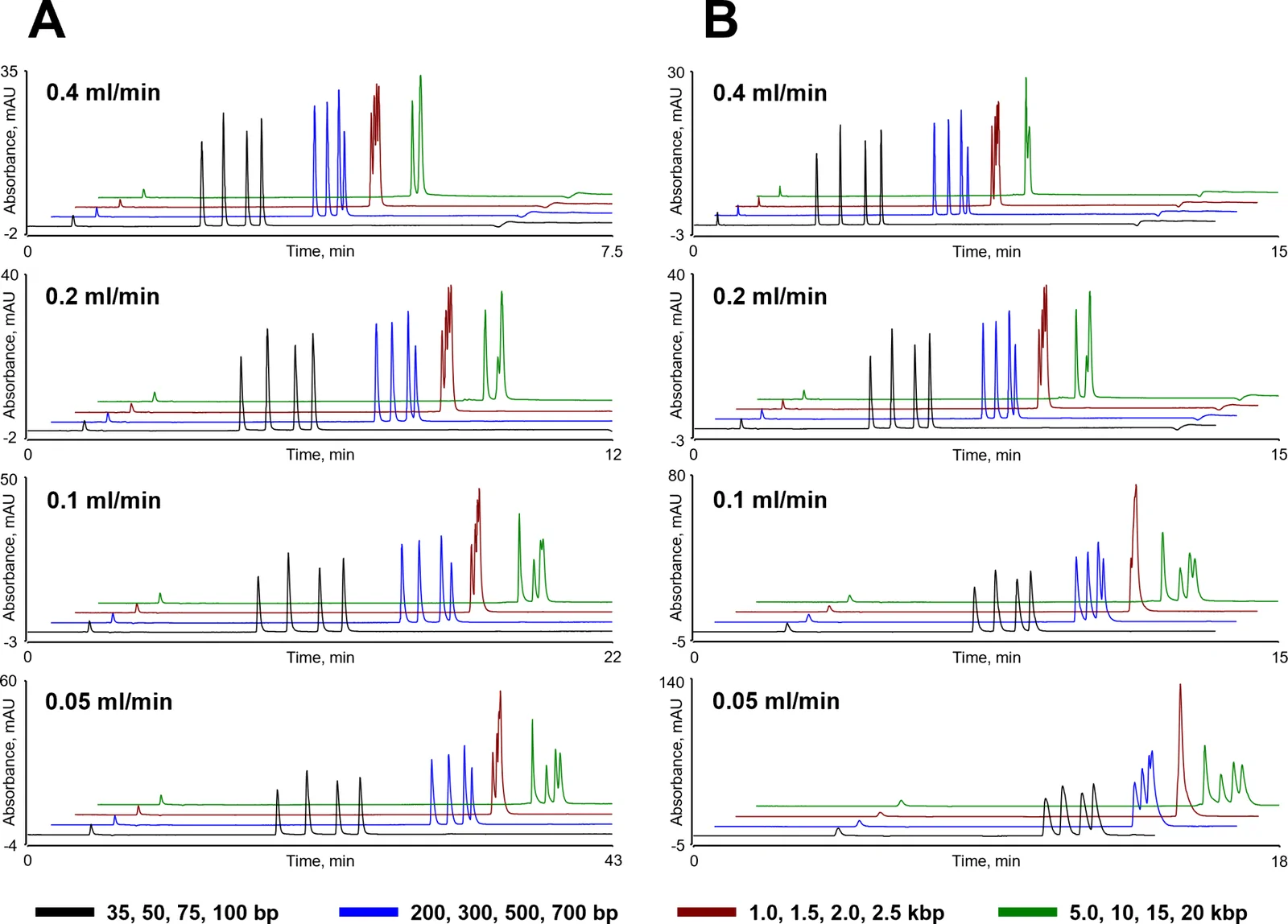
Effect of flow rate on IP-RP-HPLC separation of linear dsDNA fragments ranging from 35 bp to 20 kbp, with gradient times proportionally adjusted to match changes in flow rate (A), and with fixed gradient profiles (B). Chromatograms were acquired at flow rates of 0.05, 0.1, 0.2, and 0.4 mL/min with a 2.1 × 100 mm column. Black and blue chromatograms represent 35–100 and 200–700 bp, while brown and green represent 1.0–2.5 and 5.0–20 kbp dsDNA ladders. Mobile phase A was 100 mM TEAA (pH 7.0), and mobile phase B was ACN. The injection volume was 1 μL, column temperature was maintained at 30 °C, and UV detection was performed at 260 nm. A linear gradient from 5%B to 25%B was used for both experiments. Gradient times for scaled gradient separations were adjusted relative to 0.2 mL/min flow rate, whereas fixed gradient experiments were performed at 2%B per minute. All dsDNA fragments eluted in order of increasing fragment length.

In the next set of experiments, the flow rate was varied with *t*_g_ held constant. Under a fixed *t*_g_, increasing flow typically produces narrower peaks, improved *R*_s_, and higher *P* values.^39^ The effect arises because the same change in solvent concentration is delivered over a larger mobile phase volume within the fixed time window, creating a shallower effective gradient. As shown in Figure 3B, the shorter ladders conform to this expectation. At higher flow rates (0.4 mL/min), *P* was 31 and 16 for the 35–100 bp and 200–700 bp ladders, respectively. Reducing flow rate caused substantial losses in resolution and increased tailing and peak broadening. At 0.05 mL/min, peak capacity for the 35–100 bp ladder dropped to 6, and separation for the 200–700 bp ladder diminished, with 500 and 700 bp fragments being barely distinguishable. The 1.0–2.5 kbp ladder again followed the short ladder behavior: at 0.1 mL/min or lower, resolution between fragments deteriorated with pronounced tailing, while higher flow produced much better separation, including near baseline resolution for the 1.0–1.5 kbp pair (*R*_s_ = 1.4). In contrast, the separation for the 5.0–20 kbp ladder progressively improved as flow decreased with *t*_g_ held fixed. With 0.4 mL/min, only the 5.0 kbp fragment was marginally resolved (*R*_s_ = 0.7), and the remaining fragments merged into a single broad feature. At 0.1 mL/min flow rate, all fragments became distinguishable with *R*_s_ values of 3.8, 1.7, and 0.7, indicating a clear gain in resolution. At 0.05 mL/min the *R*_s_ between the 5.0–10 kbp pair decreased moderately (*R*_s_ = 2.9) while resolution between the 10, 15, and 20 kbp fragments increased even further (*R*_s_ = 1.9 and 1.1, respectively), despite pronounced tailing at this flow rate. Lowering flow modestly improved resolution for the largest fragments of the ladder, with a small decrease in overall peak capacity (from 7 to 6) relative to 0.1 mL/min. Full *R*_s_ and *P* values for all flow rates are available in Supporting Information, Tables S5 and S6.

Across both approaches, reducing the flow benefited the ultralarge ladder (5.0–20 kbp). The 1.0–2.5 kbp ladder showed split behavior: the 1.0–1.5 kbp pair behaved like short ladders, while the 1.5–2.5 kbp fragments required higher flow for improved resolution in both experiments. Short ladders (35–100 and 200–700 bp) benefited from higher flow only when *t*_g_ was fixed, while under proportional *t*_g_ adjustment, performance was similar across flows with an optimum at 0.1 mL/min. Taken together, these patterns are consistent with a secondary retention mechanism emerging beyond 1.5 kbp and increasing in importance for longer dsDNA (≥5.0 kbp). The improved separation of the largest fragments at lower flow rates may reflect the substantially lower diffusion of large dsDNA molecules.^48^ As fragment length increases, slower diffusion is expected to increase the importance of mass transfer effects,^39^ making separation increasingly more sensitive to flow rate. Under these conditions, reducing the flow rate provides additional time for mass transfer, reducing the impact of mass transfer hindrance and thereby decreasing peak broadening. The resulting improvement in peak shape and resolution allows the contribution of the secondary retention mechanism to become more apparent for the large dsDNA fragments. Notably, our ultralarge ladder (5.0–20 kbp) results differ from AEX observations by F. Gritti et al.,^47^ where increasing flow improved separation for large fragments (≥5.0 kbp) – likely reflecting differences in particle morphology and chromatographic mode (AEX vs IP-RP-HPLC).

### Temperature

Further on, the effect of column temperature on IP-RP-HPLC separation of dsDNA was determined using the 2.1 × 100 mm column. Column temperature was varied from 5 to 50 °C while keeping the rest of chromatographic conditions unchanged. The temperature was not increased further, since on-column melting of dsDNA was observed at 60 °C. Full chromatograms across temperatures are provided in the Supporting Information (Figure S6).

Increasing the temperature from 5 to 50 °C resulted in modest increase in retention time across all ladders (Figure 4), with smaller shifts for shorter fragments and larger shifts for longer ones. The observed increase in retention is consistent with previous studies by C. G. Huber et al.,^21^ who proposed that temperature-induced conformational changes of dsDNA duplex, including defolding and derotation of double helix, expose more accessible backbone charge for ion-pairing. The resulting stronger ion-pair interactions increase retention for dsDNA and improve resolution between length variants. In this case, the effect is more pronounced for longer fragments, which likely unfold to a greater extent and thus expose more negative charge to the mobile phase. In addition, elevated temperature also improves mass-transfer kinetics by reducing mobile phase viscosity and increasing diffusion, which further contributes to the improved chromatographic performance observed for dsDNA.^39^ Although elevated temperature typically decreases retention in IP-RP-HPLC by weakening the hydrophobic adsorption of both IPR and NA on stationary phase surface,^49^ the observed increase in retention at higher temperature suggests that enhanced electrostatic retention forces^49^ and greater accessible charge density outweighs those losses.

**Figure 4:**
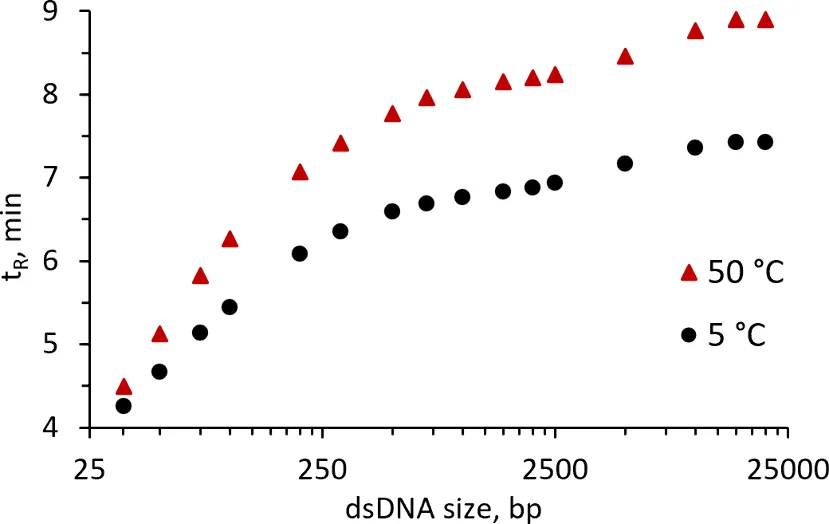
Effect of temperature on retention of linear dsDNA fragments ranging from 35 bp to 20 kbp. Retention times measured at 5 °C (black) and 50 °C (red) are shown. Separations were performed on a 2.1 × 100 mm column, using 100 mM TEAA (pH 7.0) as mobile phase A and ACN as mobile phase B. A linear gradient from 5% to 25%B at 2% B per minute was employed at a flow rate of 0.2 mL/min. Injection volume was 1 μL and UV detection was performed at 260 nm.

At elevated temperatures, peak shape and separation improved for the 35–100 bp, 200–700 bp, and 5.0–20 kbp ladders. For the shorter ladders (35–100 bp and 200–700 bp), *P* increased from 15 and 9 at 5 °C to 23 and 12 at 50 °C, respectively. For the 5.0–20 kbp ladder, at 5 °C only the 5.0 kbp fragment was resolved (*R*_s_ = 2.2) from rest of the ladder, whereas at 50 °C the 10 kbp fragment was also separated, both from 5.0 kbp (*R*_s_ = 3.7) and 15/20 kbp (*R*_s_ = 1.2), which remained coeluted as a single peak. The 1.0–2.5 kbp ladder showed mixed temperature dependence. Raising temperature from 5 to 50 °C improved *R*_s_ from 0.9 to 1.2 for the 1.0 and 1.5 kbp pair. In contrast, separation between 1.5 and 2.0 kbp and 2.0–2.5 kbp was slightly better at lower temperatures, with 10 °C providing the highest resolution for these pairs (*R*_s_ = 0.7 for both). *P* and *R*_s_ values under different column temperatures are presented in Supporting Information, Table S7.

Overall, 50 °C provided the best chromatographic performance across the investigated dsDNA size range, resulting in the highest retention and generally the best resolution for most fragment pairs. This observation is consistent with previous reports for shorter dsDNA fragments,^21,37^ and demonstrates that similar temperature trends extend to substantially larger dsDNA molecules (≥5.0 kbp). At the same time, heterogeneous behavior within the 1.0–2.5 kbp ladder may reflect the influence of additional retention contribution under these conditions.

### Ion-Pairing Reagent

In the final set of experiments, the influence of IPR strength was assessed using 2.1 × 100 mm column under chromatographic conditions identical to those used previously. Three different IPR buffers at concentration of 100 mM (pH 7.0) were tested: butylamine acetate (BAA), triethylamine acetate and dimethylamine acetate (DEAA). In addition, 100 mM ammonium acetate (unadjusted pH) was also evaluated as mobile phase A. Stronger IPRs such as hexylamine or dibutylamine were not tested, because the higher acetonitrile content required to elute large dsDNA increases risk of strand melting. *P* and *R*_s_ values with different IPRs are provided in Supporting Information, Table S8.

Previous studies have shown that retention of dsDNA in IP-RP-HPLC is predominantly governed by fragment length, although sequence composition and DNA structure can also influence chromatographic behavior.^50,51^ The GC content of the dsDNA fragments used in this study ranged from 42.2 to 62.9% and is provided in Supporting Information, Table S1. Based on nucleotide hydrophobicity,^52^ AT-rich sequences of identical length would be expected to exhibit greater retention than GC-rich sequences. Therefore, the use of stronger and more hydrophobic IPRs should reduce the influence of differences in sequence composition^24^ and provide more accurate sizing. As shown in Figure 5, stronger IPRs increased retention across all dsDNA ladders, whereas 100 mM AmAc resulted in elution at void volume (0.23 mL) with little to no separation for all ladders except 5.0–20 kbp. The monodisperse supermacroporous PS-DVB stationary phase used in this study possesses a broad pore size distribution, with pore sizes ranging from 20 to 4000 Å. Consequently, the elution order of unretained molecules reflects differences in pore accessibility. Smaller fragments have access to more pores and thus exhibit longer retention, while larger fragments access fewer pores and elute earlier. This trend was evident for the 35–100 bp, 200–700 bp, and 1.0–2.5 kbp ladders (retention times of 1.11 min, 0.94–1.04 and 0.91 min, respectively). In contrast, the 5.0–20 kbp ladder showed measurable retention with AmAc (0.97–1.3 min across fragments) and a size-ordered elution from smallest to the largest fragment (Figure 6), indicating that obstacle-navigation retention becomes the dominant mechanism for longer fragments (≥5.0 kbp) when IP-RP-HPLC retention is absent.

**Figure 5:**
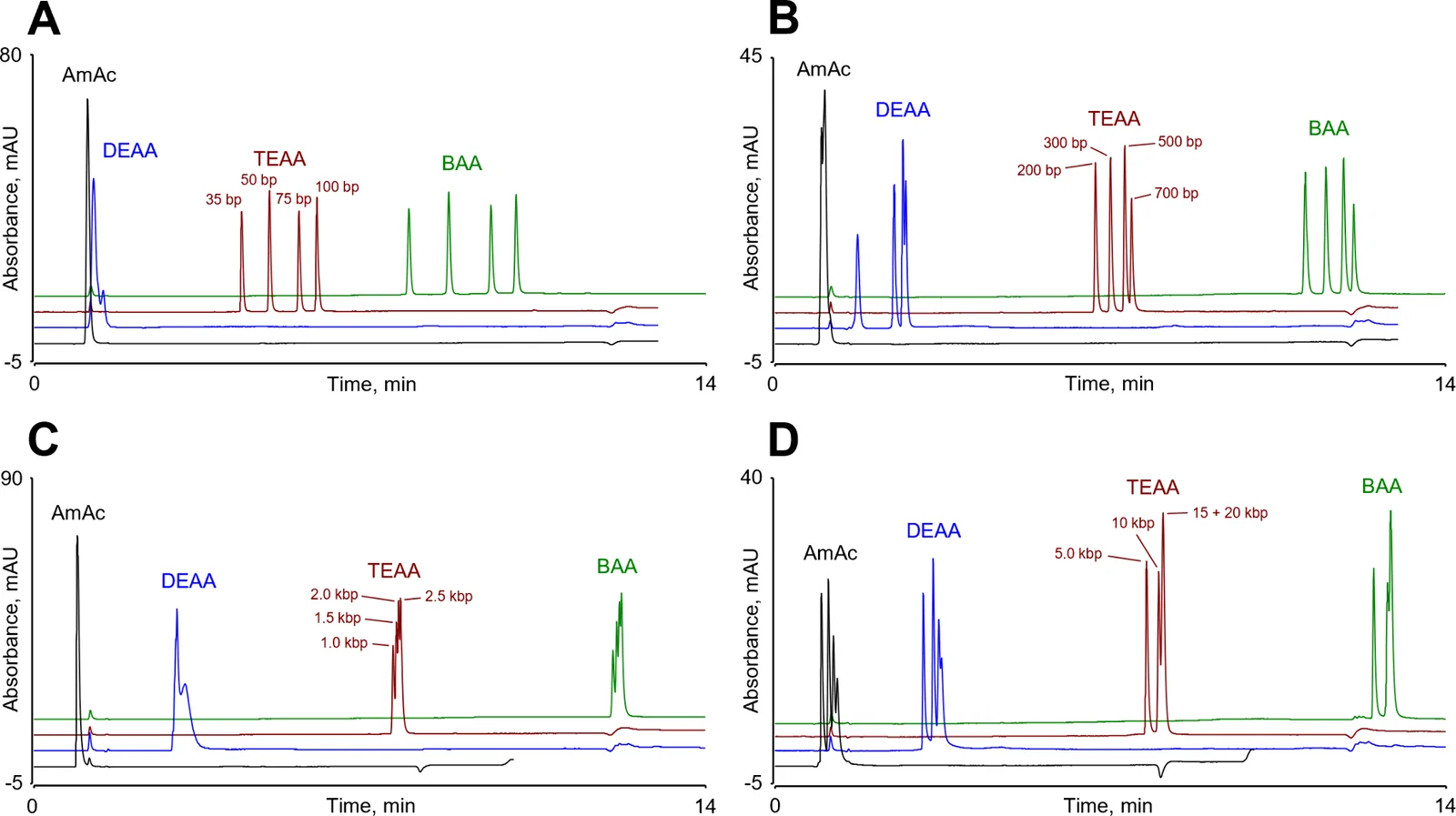
Comparison of diethylamine acetate (DEAA), triethylamine acetate (TEAA) and butylamine acetate (BAA) ion-pairing systems (100 mM, pH 7.0), and nonion-pairing ammonium acetate (AmAc) buffer (100 mM, pH not adjusted) for IP-RP-HPLC separation of 35–100 bp (A), 200–700 bp (B), 1.0–2.5 kbp (C) and 5.0–20 kbp (D) dsDNA ladders. Chromatograms were obtained on a 2.1 × 100 mm column with ACN as mobile phase B. Separations were performed using linear gradients starting at 5%B with a gradient steepness of 2%B per minute at 0.2 mL/min flow rate. Column temperature was kept at 30 °C and injection volume was 1 μL. UV detection was performed at 260 nm.

**Figure 6:**
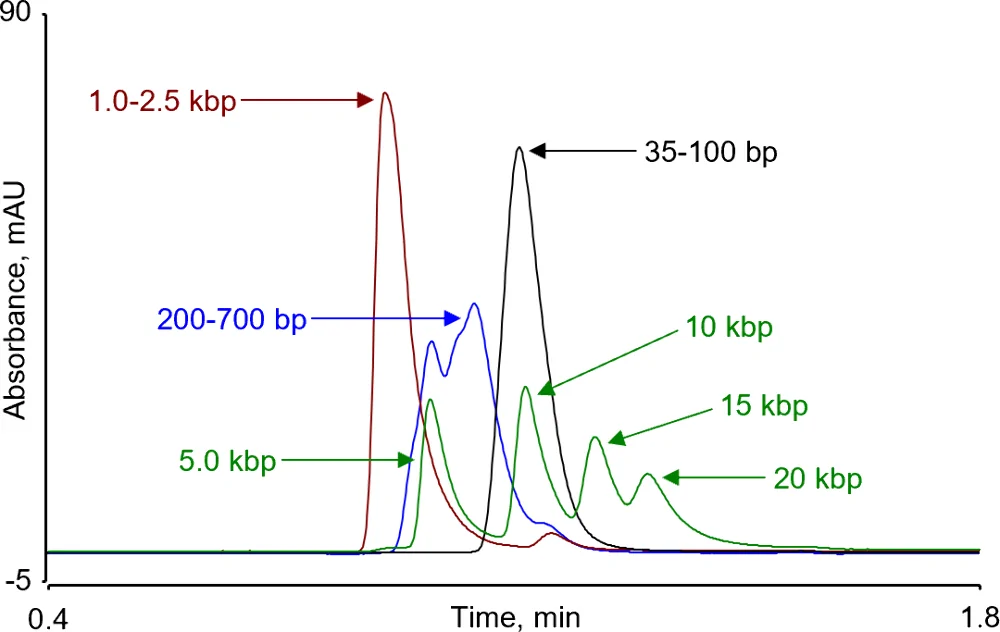
Void volume elution profiles of 35–100 bp, 200–700 bp, 1.0–2.5 kbp and 5.0–20 kbp dsDNA ladders obtained using 100 mM ammonium acetate buffer (pH not adjusted). Separations were conducted on a 2.1 × 100 mm column with ACN as mobile phase B. A linear gradient starting at 5%B with a steepness of 2%B per minute was employed. Flow rate was 0.2 mL/min, column temperature was 30 °C, and injection volume was 1 μL. UV detection was performed at 260 nm.

Beyond increased retention, stronger IPRs improved separation for the short ladders (Figure 5A,B). While DEAA produced no retention for 35–100 bp, TEAA and BAA progressively enhanced the separation. Peak capacity increased with IPR strength, reaching 25 for 35–100 bp and 11 for 200–700 bp ladder with BAA. The 1.0–2.5 kbp ladder showed similar trend (Figure 5C), with BAA yielding the highest resolutions for 1.0–1.5 kbp and 1.5–2.0 kbp (*R*_s_ = 1.0 and 0.6), whereas TEAA was slightly better for 2.0–2.5 kbp (*R*_s_ = 0.5). The 5.0–20 kbp ladder behaved differently compared to the rest of the ladders (Figure 5D). Reducing IPR strength improved separation among the largest fragments but slightly reduced resolution for the smallest pair within this ladder. In this case, pair-specific optima were observed. For 5.0–10 kbp, TEAA gave the highest resolution (*R*_s_ = 3.1), with both weaker and stronger IPRs diminishing the separation. DEAA was most optimal for resolving 10–15 kbp fragments (*R*_s_ = 1.3). AmAc yielded the best resolution for the 15–20 kbp pair (*R*_s_ = 0.8), whereas TEAA and BAA led to coelution.. Further, the effect of IPR concentration on the separation of 5.0–20 kbp was evaluated by varying TEAA from 25 to 100 mM (Figure S7). Reducing TEAA concentration did not improve separation and instead decreased resolution for all fragment pairs.

To examine how chromatographic conditions modulate IPR strength effects, 5.0–20 kbp dsDNA ladder was re-examined under conditions found previously to benefit long dsDNA fragments. Lower flow rate (0.1 mL/min) and elevated temperature (50 °C) were tested, both individually and combined. Chromatograms under these conditions are provided in Supporting Information, Figure S8. The optimal IPR for each fragment pair varied with these conditions (Figure 7), yet a consistent pattern emerged – larger fragments benefited from weaker IPRs. Under the initial conditions (Figure 7A), TEAA yielded the highest resolution for 5.0–10 kbp pair, DEAA was optimal for 10–15 kbp, and AmAc provided the best separation for 15–20 kbp fragments. Raising the temperature to 50 °C at the same flow rate (Figure 7B) changed the optimal IPR for 5.0–10 kbp pair from TEAA to BAA, while 10–15 kbp and 15–20 kbp optima were unchanged. Lowering the flow to 0.1 mL/min at 30 °C (Figure 7C) again favored BAA for 5.0–10 kbp and DEAA remained best for 10–15 kbp pair. However, optimal IPR with these conditions shifted from AmAc toward DEAA for the 15–20 kbp pair. Under the combined setting of 0.1 mL/min and 50 °C (Figure 7D), both 5.0–10 and 10–15 kbp pairs favored TEAA, while the 15–20 kbp optimum remained unchanged, with DEAA providing the best resolution.

**Figure 7:**
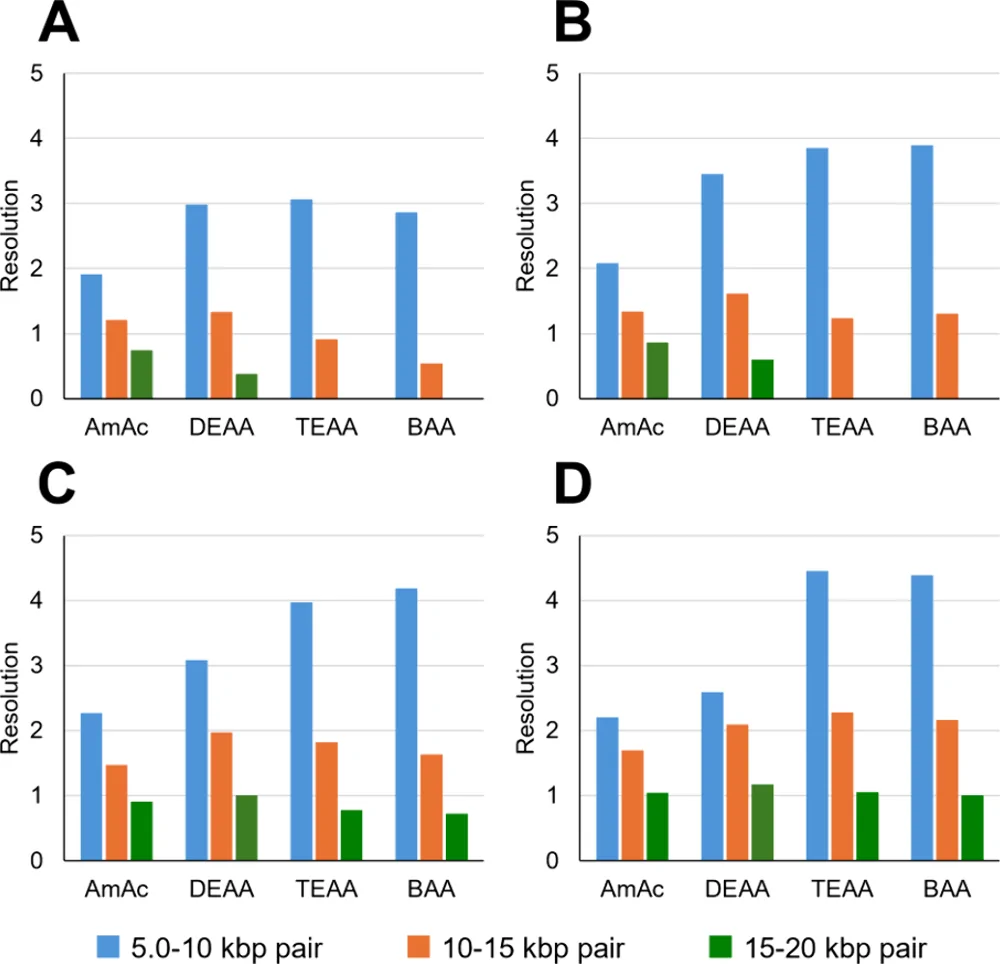
Resolution values for individual fragment pairs of 5.0–20 kbp dsDNA ladder determined on a 2.1 × 100 mm column with different ion-pairing systems under varied chromatographic conditions: 0.2 mL/min with 30 °C (A), 0.2 mL/min with 50 °C (B), 0.1 mL/min with 30 °C (C), and 0.1 mL/min with 50 °C (D).

Overall, the data show that smaller dsDNA ladders behaved as expected, with stronger IPRs increasing retention and improving resolution. In contrast, weaker IPRs generally provided better separation for the largest dsDNA fragments (≥5.0 kbp), although no single ion-pairing reagent was universally optimal across all large-fragment pairs. This behavior suggests that ion-pairing strength influences not only overall retention, but also the extent to which the secondary retention contribution affects separation. For large dsDNA fragments, ion-pairing appears to determine the affinity of the dsDNA for the stationary phase, whereas the proposed obstacle-navigation mechanism contributes to the size-based selectivity. Stronger IPRs increase retention, but may also broaden the mobile-phase composition range over which large dsDNA fragments are released from the stationary phase.^35^ Similar to the effect observed with shallower gradients, prolonged residence within this transition window may reduce the apparent size-dependent selectivity of the largest fragments. Under weaker IPR conditions, release from the stationary phase may occur over a narrower mobile phase composition range, allowing the contribution of the secondary retention behavior to become more apparent. Therefore, IPR selection should be made case by case, considering dsDNA length, desired retention, and the chromatographic conditions (temperature and flow rate).

## Conclusion

In this work, we have systematically investigated the IP-RP-HPLC behavior of linear dsDNA across a broad size range spanning 35 bp to 20 kbp using a monodisperse supermacroporous PS-DVB polymer resin. Two distinct chromatographic behaviors, separated by a practical length threshold near 1.5 kbp, were observed. Below this threshold, fragments followed classical on–off IP-RP-HPLC behavior. Increasing column length with proportional gradient time extension improved *R*_s_ and *P*, while under fixed gradients, shorter columns and higher flow rates enhanced the separation. Elevated temperatures and stronger ion-pairing reagents increased retention and improved length-based separation for short fragments. Beyond ∼1.5 kbp, chromatographic behavior trends became less uniform, as longer fragments started to deviate from conventional IP-RP-HPLC trends. For fragments larger than 5.0 kbp, longer columns, steeper gradients and lower flow rates progressively improved resolution. In contrast to shorter dsDNA fragments, weaker ion-pairing conditions provided superior separation for the largest fragments. Furthermore, measurable size-ordered retention observed under ammonium acetate conditions suggests that a secondary size-dependent retention contribution remains active even when classical IP-RP-HPLC retention is absent.

Together, these findings provide a practical framework for IP-RP-HPLC method development for linear dsDNA and demonstrate that chromatographic principles established for short nucleic acids cannot be directly extrapolated to very large dsDNA molecules. Although a dedicated mechanistic study lies outside the scope of this work, the observed trends are consistent with the presence of an additional size-dependent retention contribution beyond 1.5 kbp that becomes increasingly important with an increasing dsDNA length. These findings further highlight the importance of considering pore accessibility, additional size-dependent retention, and mass transfer effects when developing separation methods for large dsDNA fragments.

## Supplementary Material



## Notes

Contour lengths, radii of gyration, and GC/AT content of investigated dsDNA fragments; Characterization of the stationary phase by SEM, MIP, and nitrogen adsorption porosimetry; *P* and *R*_s_ values for all double-stranded DNA ladders; Chromatograms with and without postcolumn restrictor capillary; Chromatograms comparing TEAA in the aqueous mobile phase only and in both mobile phases; 5.0–20 kbp chromatograms with and without an initial isocratic hold; Chromatograms at different column temperatures; Chromatograms at varying ion-pairing reagent concentrations; 5.0–20 kbp ladder chromatograms with various ion-pairing reagents under different chromatographic conditions (PDF)
