# Supplementary material for: Length-Dependent Behavior of Linear dsDNA in IP-RP-HPLC on Supermacroporous Polymer Resin
Source: Anal Chem. 2026 Sep 8;98(36):26474–85. doi: 10.1021/acs.analchem.6c02096 (PMC13584626; doi:10.1021/acs.analchem.6c02096)
Supplement: Supplementary file 1 [file ac6c02096_si_001.pdf]

# Supporting Information

# Length-Dependent Behavior of Linear dsDNA in IP-RP-HPLC on Supermacroporous Polymer Resin

Simonas Balčiūnas[1,2,*], Audrius Žvirblis[2], Matas Damonskis[2], Frank Steiner[3], Evaldas Naujalis[1,4], Lukas Taujenis[2]

[1]Department of Analytical and Environmental Chemistry, Vilnius University, Naugarduko 24, LT03225 Vilnius, Lithuania
[2]Thermo Fisher Scientific Baltics, V. A. Graičiūno 8, LT-02241, Vilnius, Lithuania
[3]Thermo Fisher Scientific, Dornierstraße 4, 82110, Germering, Germany
[4]State Research Institute Center for Physical Sciences and Technology, Saulėtekio av. 3, LT-10257 Vilnius, Lithuania

## TABLE OF CONTENTS

**Table S1.** GC content of dsDNA fragments ........ S2
**Figure S1.** SEM image of SurePac Oligo RP MDi supermacroporous PS-DVB particles ........ S3
**Figure S2.** Pore size characterization of the monodisperse supermacroporous PS-DVB stationary phase ........ S4
**Table S2.** Contour lengths and radii of gyration of dsDNA fragments ........ S5
**Table S3.** P and $R_s$ values acquired with different column lengths using adjusted gradient profiles ........ S6
**Table S4.** P and $R_s$ values acquired with different column lengths using fixed gradient profiles ........ S7
**Figure S3.** Pressure impact on retention of dsDNA ladders ........ S8
**Figure S4.** Comparison of TEAA in the aqueous mobile phase only and in both mobile phases ........ S9
**Figure S5.** Effect of a 5 min isocratic hold on separation of the 5.0-20 kbp dsDNA ladder ........ S10
**Table S5.** P and $R_s$ values obtained with different flow rates using adjusted gradient profiles ........ S11
**Table S6.** P and $R_s$ values obtained with different flow rates using fixed gradient profiles ........ S12
**Figure S6.** Temperature influence on IP-RP-HPLC retention of dsDNA ladders ........ S13
**Table S7.** P and $R_s$ values obtained at different column temperatures ........ S14
**Table S8.** P and $R_s$ values obtained with different ion-pairing reagents ........ S15
**Figure S7.** Effect of triethylamine acetate concentration on separation of 5.0-20 kbp dsDNA ladder ........ S16
**Figure S8.** Comparison of ion-pairing reagents for separation of the 5.0-20 kbp dsDNA ladder ........ S17

**Table S1.** GC content of dsDNA fragments used in the study.

| Fragment size, bp | GC% | AT% |
|---|---|---|
| 35 | 62.9 | 37.1 |
| 50 | 62.0 | 38.0 |
| 75 | 62.7 | 37.3 |
| 100 | 59.0 | 41.0 |
| 200 | 56.5 | 43.5 |
| 300 | 54.0 | 46.0 |
| 500 | 55.0 | 45.0 |
| 700 | 46.2 | 53.8 |
| 1000 | 46.1 | 53.9 |
| 1500 | 50.2 | 49.8 |
| 2000 | 49.7 | 50.3 |
| 2500 | 51.8 | 48.2 |
| 5000 | 48.6 | 51.4 |
| 10000 | 42.2 | 57.8 |
| 15000 | 47.1 | 52.9 |
| 20000 | 47.8 | 52.2 |

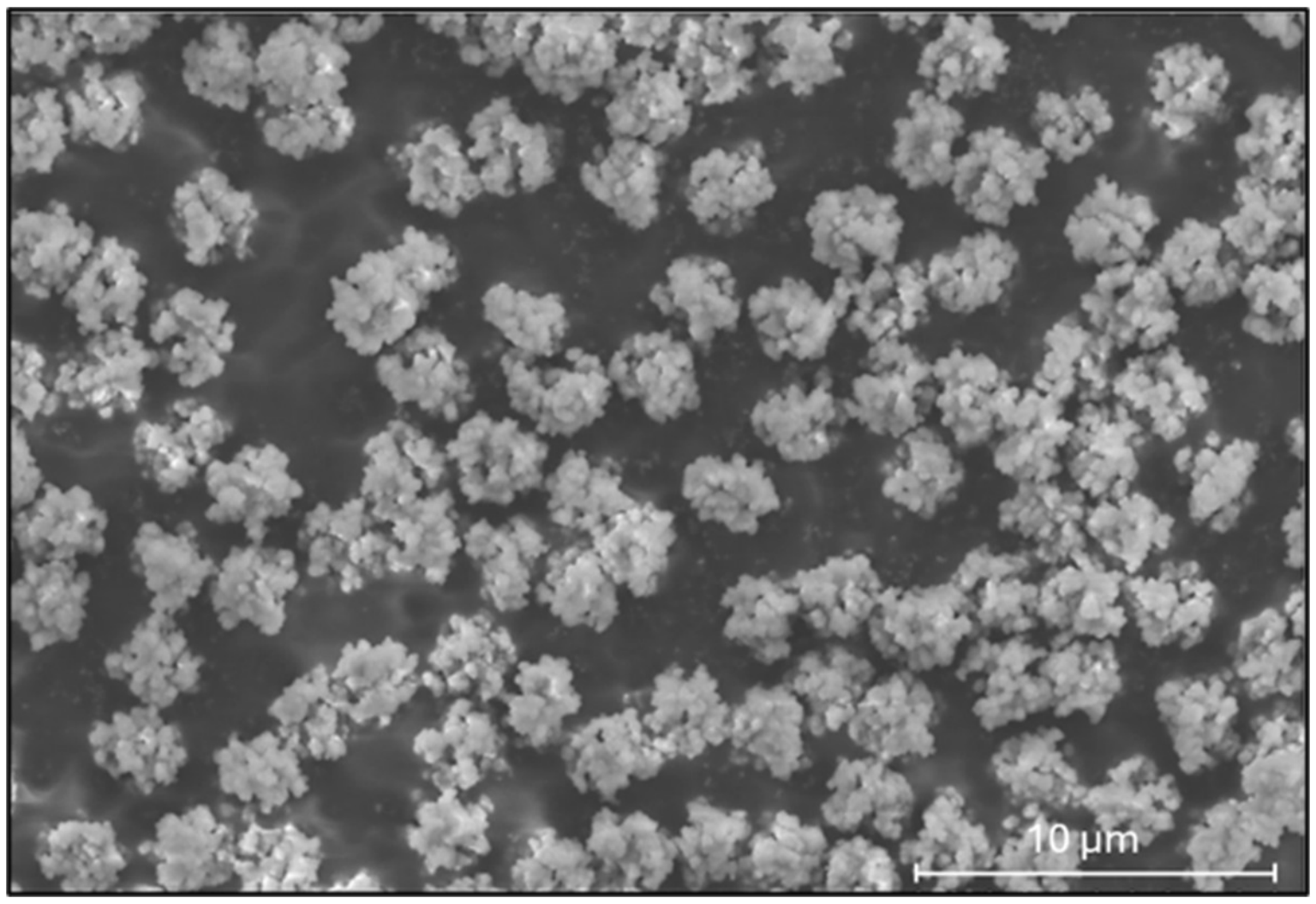


**Figure S1**. Representative SEM image of SurePac Oligo RP MDi supermacroporous PS-DVB particles, showing a rough, popcorn-like morphology and an open surface structure.

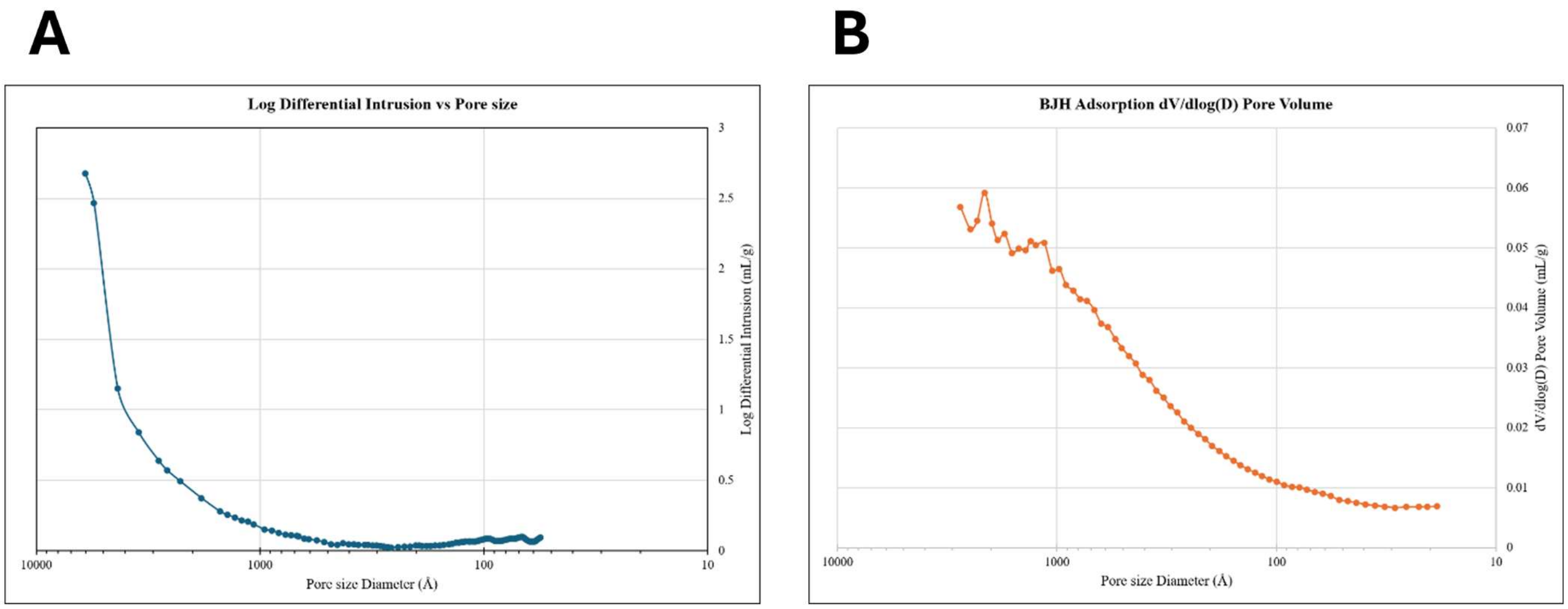


**Figure S2**. Pore size characterization of the monodisperse supermacroporous PS-DVB stationary phase by mercury intrusion porosimetry (A) and nitrogen adsorption porosimetry (B). Mercury intrusion porosimetry shows a broad apparent macropore-accessible range, with a change in slope near 4000 Å that may indicate an approximate transition between interparticle void filling and intrusion into the largest intraparticle macropores. BJH pore volume distribution derived from nitrogen adsorption data shows the contribution of smaller pores. BJH dV/dlog(D) values were smoothed using a moving-average window for visualization.

**Table S2.** Calculated contour lengths and radii of gyration of the investigated dsDNA fragments.

| Fragment size, bp | $L_c$, µm | $L_c$, Å | $R_g$, µm | $R_g$, Å |
|---|---|---|---|---|
| 35 | 0.012 | 119 | 0.009 | 93 |
| 50 | 0.017 | 170 | 0.011 | 114 |
| 75 | 0.026 | 255 | 0.014 | 143 |
| 100 | 0.034 | 340 | 0.017 | 169 |
| 200 | 0.068 | 680 | 0.025 | 251 |
| 300 | 0.102 | 1020 | 0.032 | 316 |
| 500 | 0.170 | 1700 | 0.042 | 422 |
| 700 | 0.238 | 2380 | 0.051 | 512 |
| 1000 | 0.340 | 3400 | 0.063 | 627 |
| 1500 | 0.510 | 5100 | 0.079 | 790 |
| 2000 | 0.680 | 6800 | 0.093 | 931 |
| 2500 | 0.850 | 8500 | 0.106 | 1057 |
| 5000 | 1.700 | 17000 | 0.157 | 1570 |
| 10000 | 3.400 | 34000 | 0.233 | 2330 |
| 15000 | 5.100 | 51000 | 0.294 | 2936 |
| 20000 | 6.800 | 68000 | 0.346 | 3459 |

*Contour lengths ($L_c$) were calculated using 3.4 Å per base pair.

**Table S3.** Peak capacity and resolution values acquired with different column lengths using adjusted gradient profiles.

| | 20 mm | 50 mm | 100 mm | 200 mm | 300 mm |
|---|---|---|---|---|---|
| 35-100 bp dsDNA | | | | | |
| P | 10 | 20 | 33 | 43 | 49 |
| $R_s$ (35-50 bp) | 3.3 | 6.8 | 11.3 | 15.3 | 16.7 |
| $R_s$ (50-75 bp) | 3.7 | 7.3 | 12.9 | 16.1 | 20.4 |
| $R_s$ (75-100 bp) | 2.3 | 5.4 | 7.4 | 10.8 | 11.8 |
| 200-700 bp dsDNA | | | | | |
| P | 6 | 11 | 17 | 23 | 26 |
| $R_s$ (200-300 bp) | 2.2 | 4.3 | 6.4 | 8.6 | 10.1 |
| $R_s$ (300-500 bp) | 2.1 | 4.0 | 6.0 | 8.4 | 9.9 |
| $R_s$ (500-700 bp) | 1.0 | 2.0 | 3.1 | 4.4 | 4.5 |
| 1.0-2.5 kbp dsDNA | | | | | |
| P | 1 | 3 | 4 | 5 | 6 |
| $R_s$ (1.0-1.5 kbp) | 0.4 | 0.7 | 1.7 | 2.1 | 2.6 |
| $R_s$ (1.5-2.0 kbp) | - | 0.1 | 0.9 | 1.2 | 1.5 |
| $R_s$ (2.0-2.5 kbp) | - | - | 0.6 | 1.0 | 1.2 |
| 5.0-20 kbp dsDNA | | | | | |
| P | 2 | 2 | 3 | 6 | 7 |
| $R_s$ (5.0-10k bp) | 0.6 | 1.4 | 2.3 | 2.9 | 3.2 |
| $R_s$ (10-20 kbp) | - | - | - | 0.1 | 0.4 |
| $R_s$ (15-20 kbp) | - | - | - | - | - |

**Table S4.** Peak capacity and resolution values acquired with different column lengths using fixed gradient profiles.

| | 20 mm | 50 mm | 100 mm | 200 mm | 300 mm |
|---|---|---|---|---|---|
| 35-100 bp dsDNA | | | | | |
| P | 21 | 20 | 19 | 17 | 15 |
| $R_s$ (35-50 bp) | 7.0 | 6.4 | 6.0 | 5.8 | 4.9 |
| $R_s$ (50-75 bp) | 7.5 | 7.2 | 6.8 | 6.4 | 5.7 |
| $R_s$ (75-100 bp) | 5.0 | 5.2 | 5.0 | 4.0 | 3.4 |
| 200-700 bp dsDNA | | | | | |
| P | 12 | 11 | 10 | 9 | 7 |
| $R_s$ (200-300 bp) | 4.4 | 4.3 | 3.8 | 3.4 | 2.6 |
| $R_s$ (300-500 bp) | 4.1 | 4.0 | 3.5 | 3.1 | 2.3 |
| $R_s$ (500-700 bp) | 2.1 | 2.0 | 1.8 | 1.4 | 0.9 |
| 1.0-2.5 kbp dsDNA | | | | | |
| P | 2 | 3 | 4 | 3 | 3 |
| $R_s$ (1.0-1.5 kbp) | 1.1 | 1.1 | 1.0 | 0.8 | 0.6 |
| $R_s$ (1.5-2.0 kbp) | - | - | 0.7 | 0.7 | 0.7 |
| $R_s$ (2.0-2.5 kbp) | - | - | 0.6 | 0.8 | 0.8 |
| 5.0-20 kbp dsDNA | | | | | |
| P | 2 | 2 | 5 | 8 | 8 |
| $R_s$ (5.0-10k bp) | - | 1.4 | 3.0 | 4.8 | 5.3 |
| $R_s$ (10-20 kbp) | - | - | 0.8 | 1.8 | 2.3 |
| $R_s$ (15-20 kbp) | - | - | - | 0.5 | 1.0 |

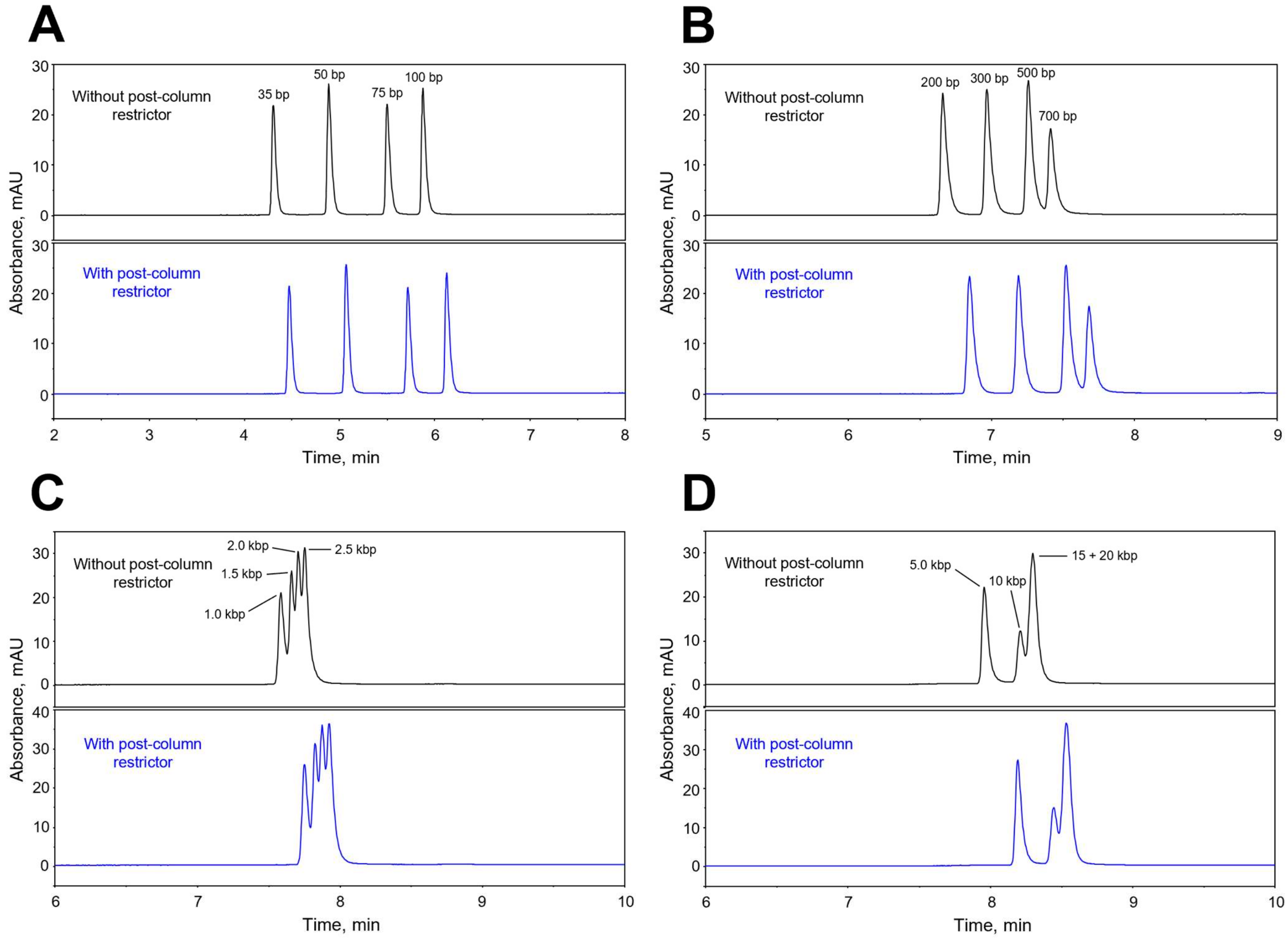


**Figure S3.** Pressure impact on retention of 35-100 bp (A), 200-700 bp (B), 1.0-2.5 kbp (C) and 5.0-20 kbp (D) dsDNA ladders. Chromatograms were obtained using 2.1 × 100 mm column at flow rate of 0.2 ml/min, with column maintained at 30 °C. 100 mM TEAA (pH 7.0) was used as mobile phase A, and ACN as mobile phase B. Separations were performed using a linear gradient from 5%B to 25%B with a steepness of 2%B per minute. The injection volume was 1 µl, and UV detection was at 260 nm. Top chromatograms (black) represent separations performed without post-column restriction capillary installed, while bottom (blue) represent separations obtained while using post-column restrictor capillary between column and detector to match the pressure of 300 mm length column of identical inner diameter.

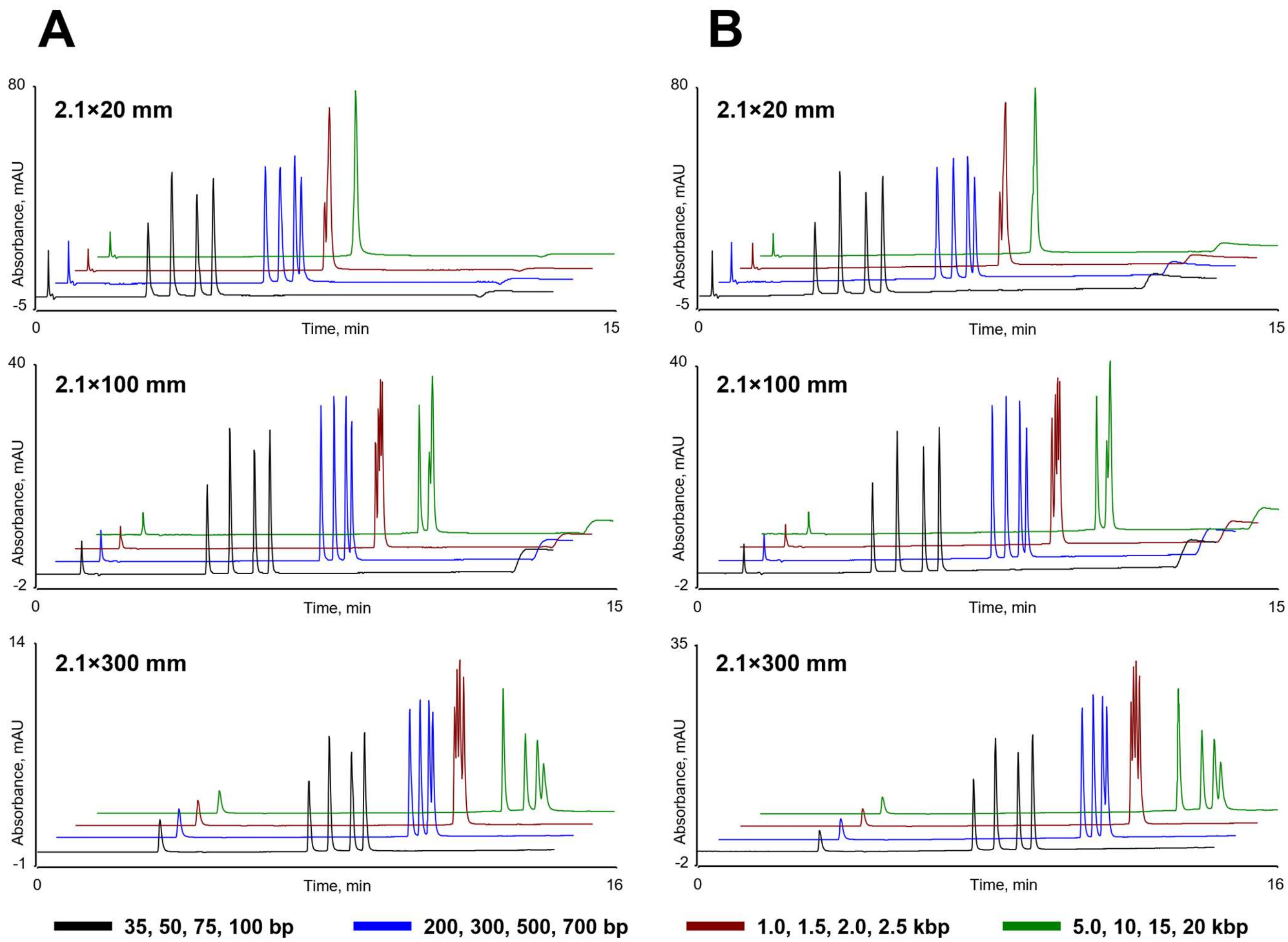


**Figure S4**. Comparison of IP-RP-HPLC separations of linear dsDNA fragments using 100 mM TEAA present only in the aqueous mobile phase (A), and in both aqueous and organic mobile phases (B). Separations were performed on 2.1 × 20 mm, 2.1 × 100 mm, and 2.1 × 300 mm columns. Black and blue chromatograms represent 35-100 and 200-700 bp, while brown and green represent 1.0-2.5 and 5.0-20 kbp dsDNA ladders. Mobile phase A consisted of 100 mM TEAA (pH 7.0), while mobile phase B was either ACN or ACN containing 100 mM TEAA. A linear gradient from 5%B to 25%B at 2%B per minute was employed. The flow rate was 0.2 mL/min, column temperature was maintained at 30 °C, injection volume was 1 µL, and UV detection was performed at 260 nm. All dsDNA fragments eluted in order of increasing fragment length.

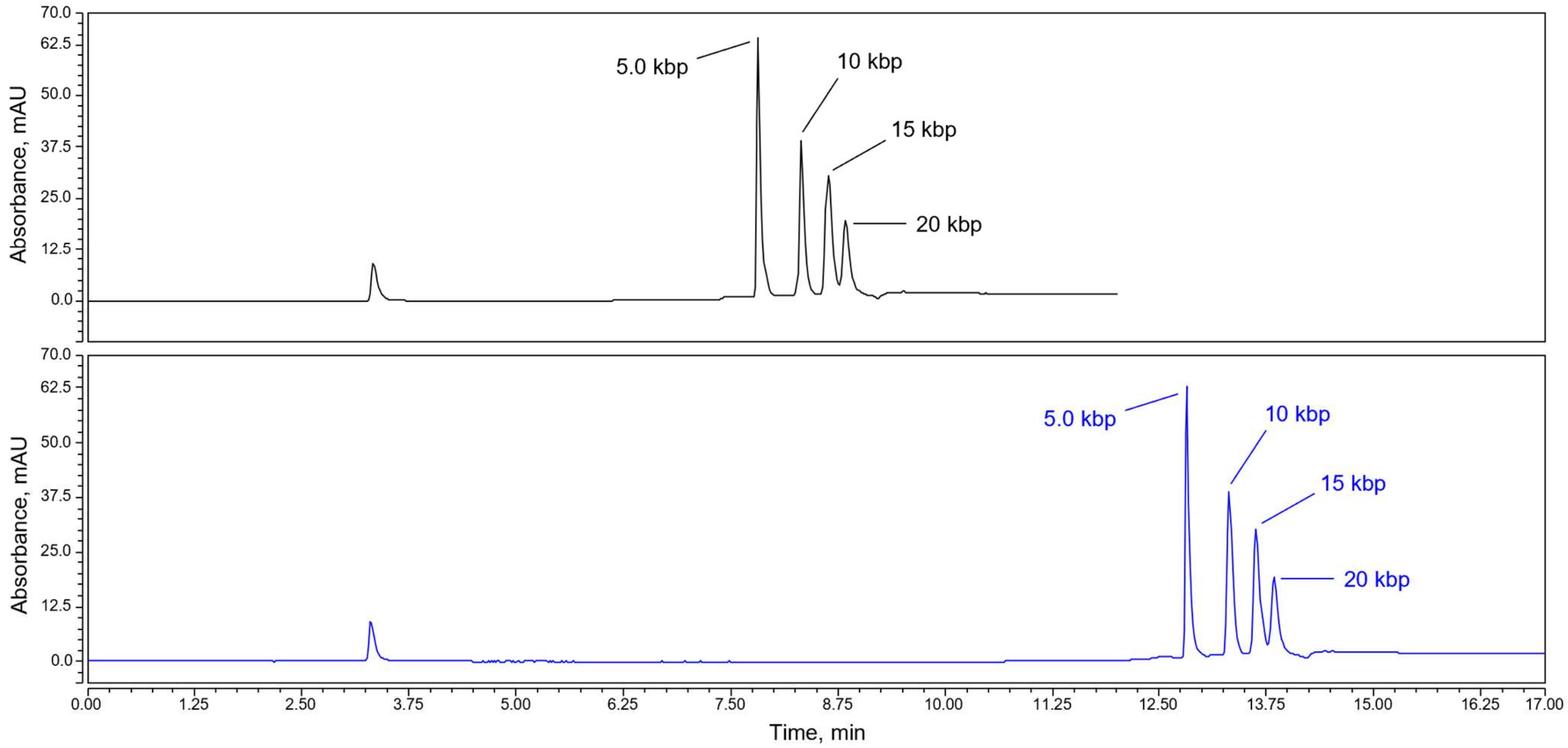


**Figure S5**. Comparison of IP-RP-HPLC separation of the 5.0–20 kbp dsDNA ladder with (A) and without (B) a 5 min isocratic hold prior to a 5 min linear gradient. Separations were performed on a 2.1 × 300 mm column. Mobile phase A was 100 mM TEAA (pH 7.0) and mobile phase B was ACN. The linear gradient was from 5%B to 25%B, the flow rate was 0.2 mL/min, column temperature was maintained at 30 °C, the injection volume was 1 μL, and UV detection was performed at 260 nm. The isocratic hold was performed at the initial mobile phase composition (5%B).

**Table S5.** Peak capacity and resolution values obtained with different flow rates using adjusted gradient profiles.

| | 0.05 ml/min | 0.1 ml/min | 0.2 ml/min | 0.4 ml/min |
|---|---|---|---|---|
| 35-100 bp dsDNA | | | | |
| P | 19 | 20 | 20 | 20 |
| $R_s$ (35-50 bp) | 6.4 | 7.1 | 6.9 | 6.8 |
| $R_s$ (50-75 bp) | 6.5 | 7.3 | 7.3 | 7.3 |
| $R_s$ (75-100 bp) | 4.6 | 4.7 | 4.6 | 4.6 |
| 200-700 bp dsDNA | | | | |
| P | 12 | 13 | 11 | 11 |
| $R_s$ (200-300 bp) | 4.2 | 4.6 | 4.1 | 4.0 |
| $R_s$ (300-500 bp) | 4.3 | 4.9 | 4.1 | 3.7 |
| $R_s$ (500-700 bp) | 2.3 | 2.4 | 2.0 | 1.8 |
| 1.0-2.5 kbp dsDNA | | | | |
| P | 3 | 3 | 3 | 3 |
| $R_s$ (1.0-1.5 kbp) | 1.1 | 1.1 | 1.0 | 0.9 |
| $R_s$ (1.5-2.0 kbp) | 0.4 | 0.5 | 0.6 | 0.7 |
| $R_s$ (2.0-2.5 kbp) | 0.3 | 0.4 | 0.6 | 0.7 |
| 5.0-20 kbp dsDNA | | | | |
| P | 8 | 7 | 4 | 3 |
| $R_s$ (5.0-10k bp) | 4.6 | 4.1 | 2.9 | 2.0 |
| $R_s$ (10-20 kbp) | 2.4 | 1.3 | 0.8 | - |
| $R_s$ (15-20 kbp) | 1.0 | 0.3 | - | - |

**Table S6.** Peak capacity and resolution values obtained with different flow rates using fixed gradient profiles.

| | 0.05 ml/min | 0.1 ml/min | 0.2 ml/min | 0.4 ml/min |
|---|---|---|---|---|
| 35-100 bp dsDNA | | | | |
| P | 6 | 11 | 20 | 31 |
| $R_s$ (35-50 bp) | 1.7 | 3.6 | 6.7 | 11.2 |
| $R_s$ (50-75 bp) | 1.9 | 3.8 | 7.2 | 12.0 |
| $R_s$ (75-100 bp) | 1.2 | 2.5 | 4.6 | 7.5 |
| 200-700 bp dsDNA | | | | |
| P | 3 | 7 | 11 | 16 |
| $R_s$ (200-300 bp) | 1.0 | 2.4 | 4.0 | 6.3 |
| $R_s$ (300-500 bp) | 0.9 | 2.2 | 4.1 | 5.8 |
| $R_s$ (500-700 bp) | 0.3 | 1.0 | 2.0 | 2.9 |
| 1.0-2.5 kbp dsDNA | | | | |
| P | 1 | 2 | 3 | 4 |
| $R_s$ (1.0-1.5 kbp) | - | 0.3 | 1.0 | 1.4 |
| $R_s$ (1.5-2.0 kbp) | - | - | 0.6 | 0.8 |
| $R_s$ (2.0-2.5 kbp) | - | - | 0.5 | 0.6 |
| 5.0-20 kbp dsDNA | | | | |
| P | 6 | 7 | 4 | 2 |
| $R_s$ (5.0-10k bp) | 2.7 | 3.7 | 2.9 | 0.5 |
| $R_s$ (10-20 kbp) | 1.8 | 1.6 | 0.8 | 0.2 |
| $R_s$ (15-20 kbp) | 1.1 | 0.7 | - | - |

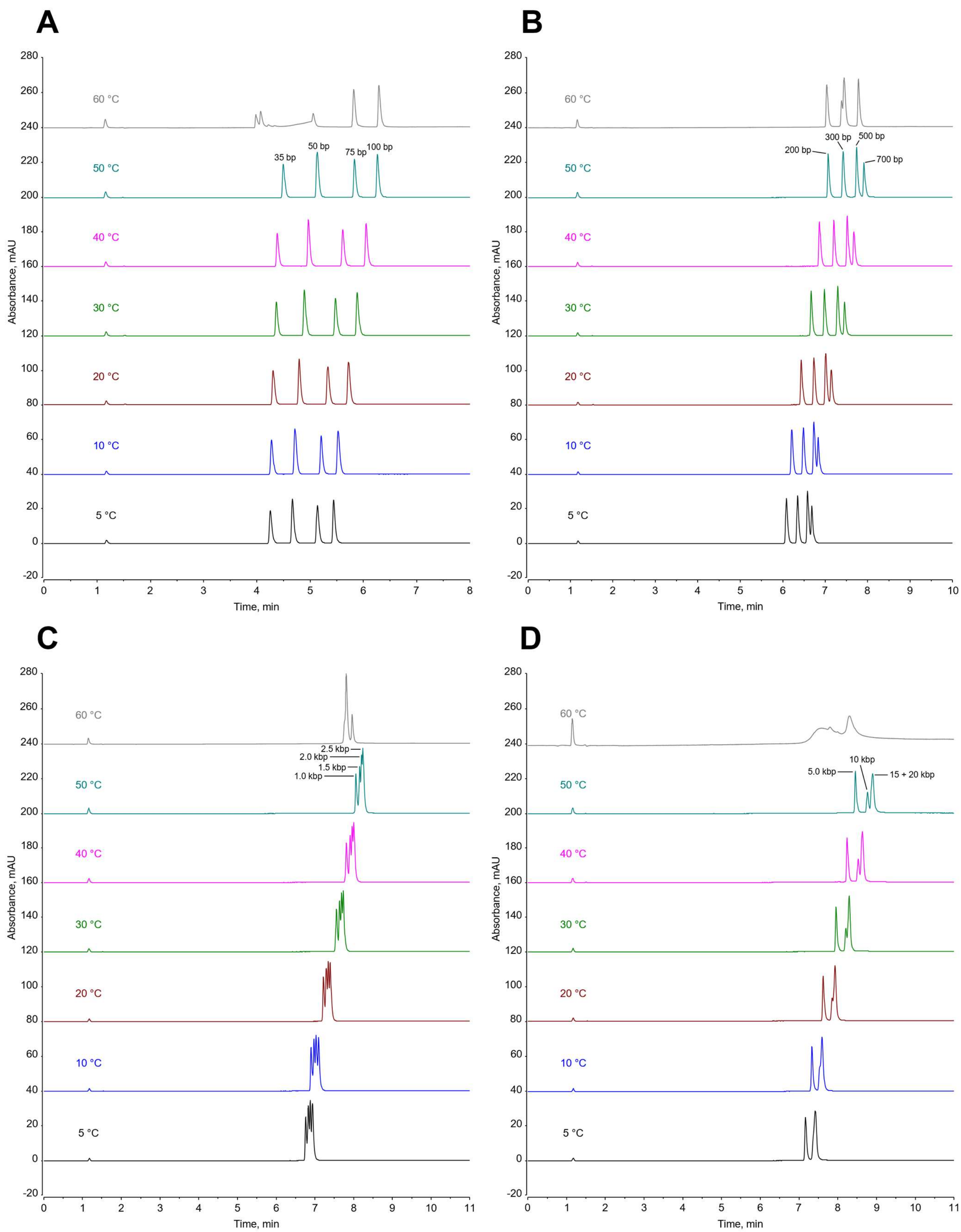


**Figure S6.** Temperature influence on IP-RP-HPLC retention of 35-100 bp (A), 200-700 bp (B), 1.0-2.5 kbp (C) and 5.0-20 kbp (D) dsDNA ladders. Chromatograms were produced with 2.1 × 100 mm column at flow rate of 0.2 ml/min, with column temperatures ranging from 5 to 50 °C. Mobile phase A was 100 mM TEAA (pH 7.0), and ACN was mobile phase B. Injection was 1 µl, UV detection was performed at 260 nm, and separations were performed using a linear gradient from 5%B to 25%B with a gradient steepness of 2%B per minute.

**Table S7.** Peak capacity and resolution values obtained at different column temperatures.

| | 5 °C | 10 °C | 20 °C | 30 °C | 40 °C | 50 °C |
|---|---|---|---|---|---|---|
| 35-100 bp dsDNA | | | | | | |
| P | 15 | 16 | 18 | 20 | 22 | 23 |
| $R_s$ (35-50 bp) | 4.9 | 5.3 | 6.1 | 6.6 | 7.4 | 7.9 |
| $R_s$ (50-75 bp) | 5.7 | 6.0 | 6.7 | 7.2 | 8.0 | 8.6 |
| $R_s$ (75-100 bp) | 3.8 | 4.0 | 4.6 | 5.0 | 5.2 | 5.3 |
| 200-700 bp dsDNA | | | | | | |
| P | 9 | 9 | 10 | 11 | 11 | 12 |
| $R_s$ (200-300 bp) | 3.3 | 3.4 | 3.8 | 4.0 | 4.3 | 4.4 |
| $R_s$ (300-500 bp) | 3.1 | 3.2 | 3.6 | 3.9 | 4.0 | 4.1 |
| $R_s$ (500-700 bp) | 1.3 | 1.4 | 1.7 | 2.0 | 2.1 | 2.4 |
| 1.0-2.5 kbp dsDNA | | | | | | |
| P | 3 | 3 | 3 | 3 | 3 | 3 |
| $R_s$ (1.0-1.5 kbp) | 0.9 | 0.9 | 1.0 | 1.1 | 1.2 | 1.2 |
| $R_s$ (1.5-2.0 kbp) | 0.7 | 0.7 | 0.6 | 0.6 | 0.6 | 0.5 |
| $R_s$ (2.0-2.5 kbp) | 0.7 | 0.7 | 0.6 | 0.5 | 0.5 | 0.4 |
| 5.0-20 kbp dsDNA | | | | | | |
| P | 4 | 4 | 5 | 5 | 6 | 6 |
| $R_s$ (5.0-10k bp) | 2.2 | 2.3 | 2.5 | 2.9 | 3.5 | 3.7 |
| $R_s$ (10-20 kbp) | - | - | 0.6 | 0.8 | 1.1 | 1.2 |
| $R_s$ (15-20 kbp) | - | - | - | - | - | - |

**Table S8.** Peak capacity and resolution values obtained with different ion-pairing reagents.

| | AmAc | DEAA | TEAA | BAA |
|---|---|---|---|---|
| 35-100 bp dsDNA | | | | |
| P | - | - | 21 | 25 |
| $R_s$ (35-50 bp) | - | - | 7.4 | 8.7 |
| $R_s$ (50-75 bp) | - | - | 7.8 | 9.2 |
| $R_s$ (75-100 bp) | - | - | 4.7 | 5.6 |
| 200-700 bp dsDNA | | | | |
| P | - | 10 | 11 | 11 |
| $R_s$ (200-300 bp) | - | 7.63 | 3.9 | 4.3 |
| $R_s$ (300-500 bp) | - | 2.33 | 3.6 | 3.8 |
| $R_s$ (500-700 bp) | - | 0.73 | 1.8 | 2.2 |
| 1.0-2.5 kbp dsDNA | | | | |
| P | - | - | 3 | 3 |
| $R_s$ (1.0-1.5 kbp) | - | - | 0.9 | 1.0 |
| $R_s$ (1.5-2.0 kbp) | - | - | 0.5 | 0.6 |
| $R_s$ (2.0-2.5 kbp) | - | - | 0.5 | 0.4 |
| 5.0-20 kbp dsDNA | | | | |
| P | 5 | 7 | 5 | 6 |
| $R_s$ (5.0-10k bp) | 1.9 | 3.0 | 3.1 | 2.9 |
| $R_s$ (10-20 kbp) | 1.2 | 1.3 | 0.9 | 0.5 |
| $R_s$ (15-20 kbp) | 0.8 | 0.4 | - | - |

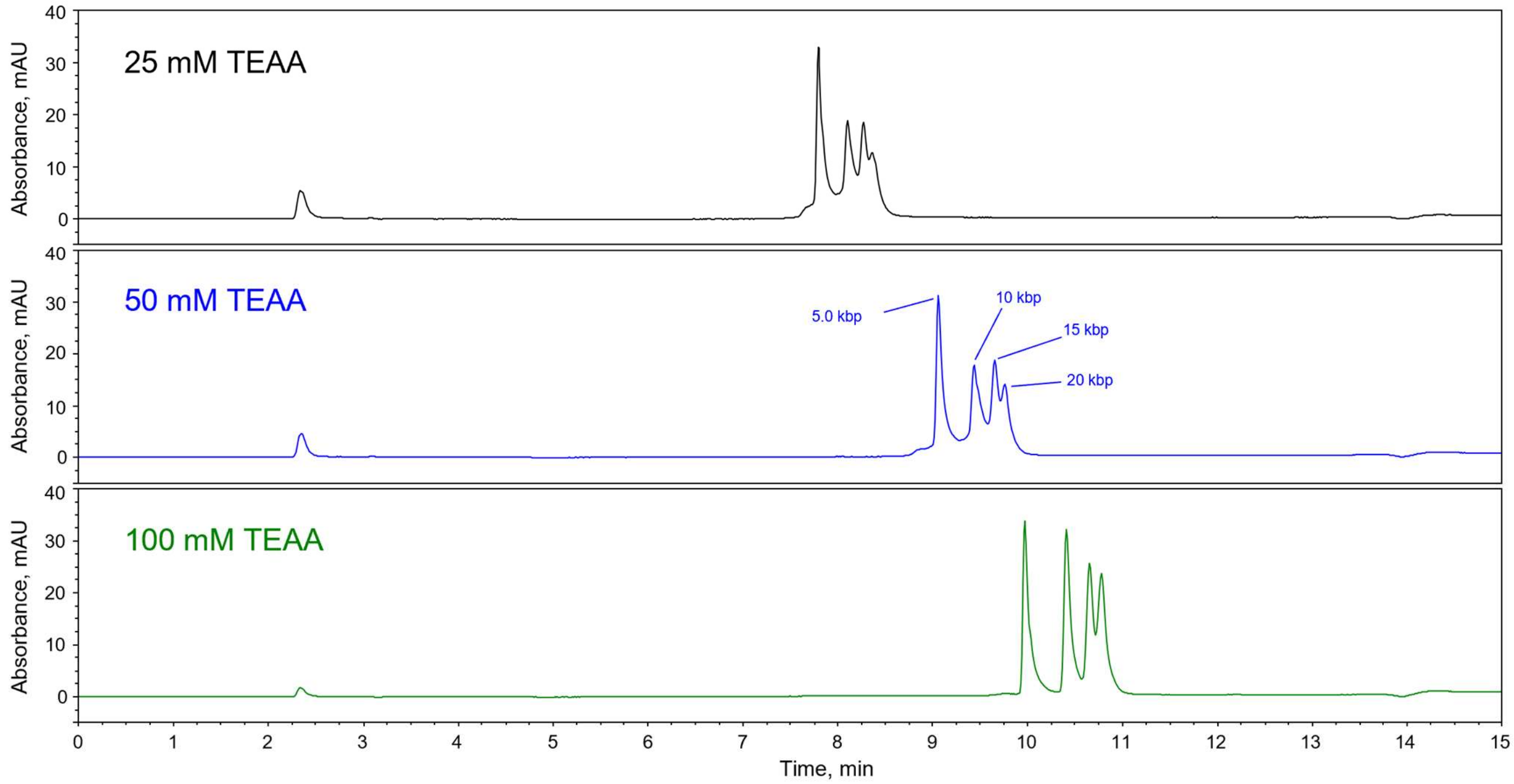


**Figure S7.** Effect of triethylamine acetate (TEAA) concentration on IP-RP-HPLC separation of 5.0-20 kbp dsDNA ladder. Chromatograms were obtained with TEAA concentrations of 25 mM (A), 50 mM (B) and 100 mM (C) using a 2.1 × 100 mm column. ACN was used as mobile phase B. Flow rate was 0.1 ml/min, column temperature was 30 °C, injection volume was 1 µl, and UV detection was performed at 260 nm. Separations were performed using a linear gradient starting at 5%B with a gradient steepness of 2%B per minute.

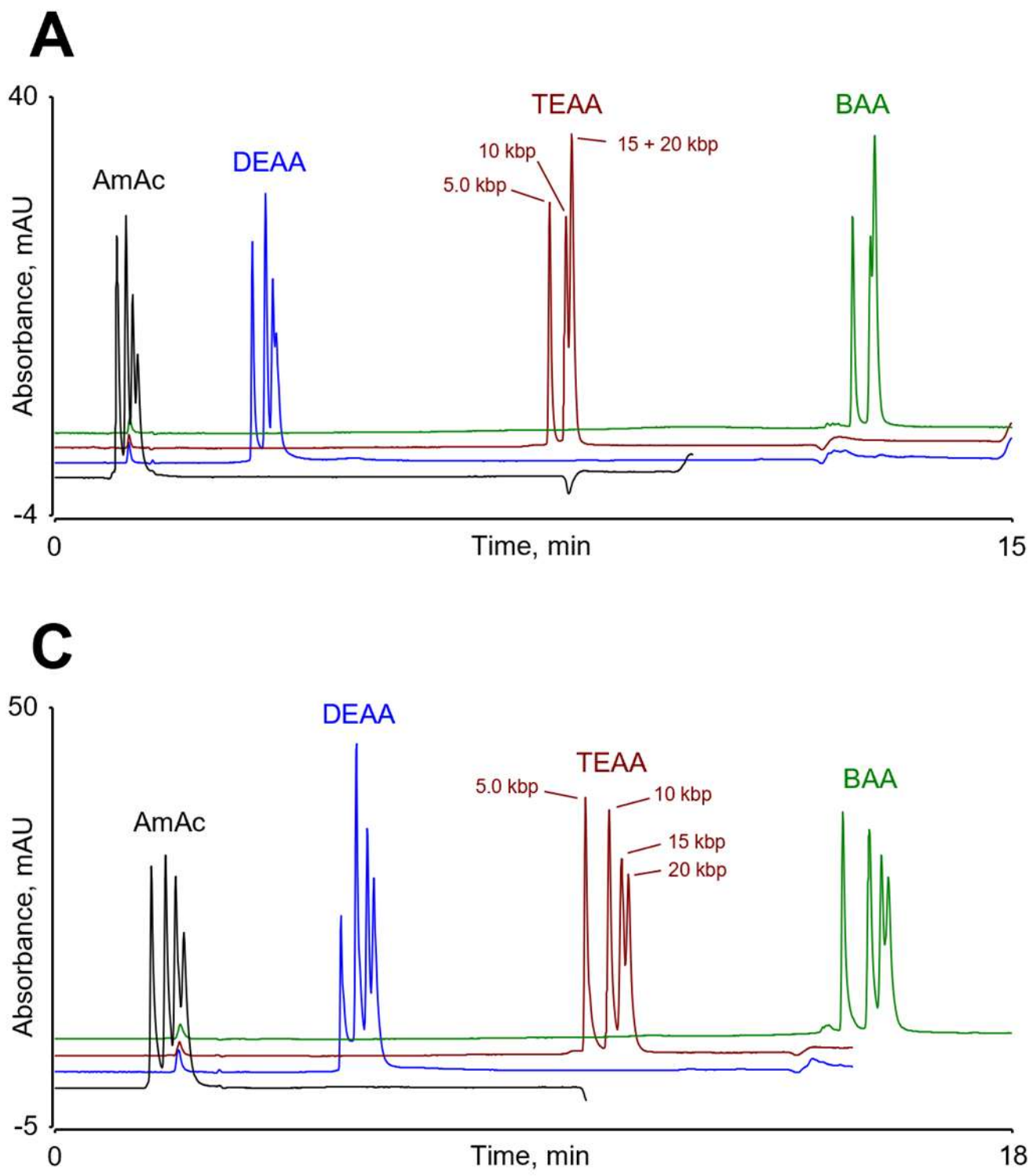


**Figure S8.** Comparison of diethylamine acetate (DEAA), triethylamine acetate (TEAA) and butylamine acetate (BAA) ion-pairing systems (100 mM, pH 7.0), and non-ion-pairing ammonium acetate (AmAc) buffer (100 mM, pH not adjusted) for IP-RP-HPLC separation of 5.0-20 kbp dsDNA ladder at 0.2 ml/min with 30 °C (A), 0.2 ml/min with 50 °C (B), 0.1 ml/min with 30 °C (C) and 0.1 ml/min with 50 °C (D). Chromatograms were obtained on a 2.1 × 100 mm column with ACN as mobile phase B. Separations were performed using a linear gradient starting at 5%B with a gradient steepness of 2%B per minute at 0.2 ml/min flow rate. Column temperature was kept at 30 °C, injection volume was 1 µL and UV detection was performed at 260 nm.
